# Differential gene regulation by a synthetic vitamin D receptor ligand and active vitamin D in human cells

**DOI:** 10.1371/journal.pone.0295288

**Published:** 2023-12-13

**Authors:** Miho Iwaki, Yoshiaki Kanemoto, Takahiro Sawada, Koki Nojiri, Tomohiro Kurokawa, Rino Tsutsumi, Kazuo Nagasawa, Shigeaki Kato

**Affiliations:** 1 Graduate School of Technology, Tokyo University of Agriculture and Technology, Koganei, Tokyo, Japan; 2 Graduate School of Life Science and Technology, Iryo Sosei University, Iino, Chuo-dai, Iwaki, Fukushima, Japan; 3 Research Institute of Innovative Medicine, Tokiwa Foundation, Iwaki, Fukushima, Japan; 4 School of Medicine, Fukushima Medical University, Fukushima, Japan; Augusta University, UNITED STATES

## Abstract

Vitamin D (VD) exerts a wide variety of biological functions including calcemic activity. VD nutritional status is closely associated with the onset and development of chronic diseases. To develop a VD analog with the desired VD activity but without calcemic activity, we screened synthetic VDR antagonists. We identified 1α,25-dihydroxyvitamin D_3_-26-23-lactams (DLAM)-**2a**-**d** (DLAM-**2**s) as nuclear vitamin D receptor (VDR) ligands in a competitive VDR binding assay for 1α,25(OH)_2_ vitamin D_3_ (1α,25(OH)_2_D_3_), and DLAM-**2**s showed an antagonistic effect on 1α,25(OH)_2_ D_3_-induced cell differentiation in HL60 cells. In a luciferase reporter assay in which human VDR was exogenously expressed in cultured COS-1 cells, DLAM-2s acted as transcriptional antagonists. Consistently, DLAM-**2**s had an antagonistic effect on the 1α,25(OH)_2_D_3_-induced expression of a known VD target gene [*Cytochrome P450 24A1 (CYP24A1)*], and VDR bound DLAM-**2**s was recruited to an endogenous VD response element in chromatin in human keratinocytes (HaCaT cells) endogenously expressing VDR. In an ATAC-seq assay, the effects of 1α,25(OH)_2_ D_3_ and DLAM-**2b** on chromatin reorganization were undetectable in HaCaT cells, while the effect of an androgen receptor (AR) antagonist (bicalutamide) was confirmed in prostate cancer cells (LNCaP) expressing endogenous AR. However, whole genome analysis using RNA-seq and ATAC (Assay for Transposase Accessible Chromatin)-seq revealed differential gene expression profiles regulated by DLAM-**2b** versus 1α,25(OH)_2_D_3_. The upregulated and downregulated genes only partially overlapped between cells treated with 1α,25(OH)_2_D_3_ and those treated with DLAM-**2b**. Thus, the present findings illustrate a novel VDR ligand with gene regulatory activity differing from that of 1α,25(OH)_2_D_3_.

## Introduction

Vitamin D (VD) is a major hormone controlling calcium homeostasis [1,2]. In addition to its calcemic actions, VD regulates a number of biological events under physiological conditions [3,4]. In the pathological setting, the onset and development of many non-communicable chronic diseases such as cancer are closely related to nutritious VD deficiency, and hence adequate intake of VD from diet is strongly recommended, especially in the elderly [5,6]. VD acts similarly to steroid/thyroid hormones; however, unlike these hormones, VD is derived from diet as a fat-soluble vitamin in addition to its endogenous production from precursors [7,8]. The most active form of VD is 1α,25-dihydroxyvitamin D_3_ [1α,25(OH)_2_D_3_], and its biosynthesis requires two major P450 enzymes. The most abundant VD precursor in serum is vitamin D_3_, which is first hydroxylated into 25(OH)_2_D_3_ by hepatic CYP2R1. Upon increased demand of 1α,25(OH)_2_D_3_ in the body, 25(OH)D_3_ circulating in serum is then hydroxylated into 1α,25(OH)_2_D_3_ by CYP27B1, which is expressed mainly in the proximal tubules in the kidney as well as in skin [9]. The renal expression of *CYP27B1* is reciprocally regulated by two calcemic hormones, parathyroid hormone (a positive regulator) and 1α,25 (OH)_2_D_3_ (a negative feedback regulator), and *CYP27B1* tightly regulates the production of the appropriate amount of 1α,25(OH)_2_D_3_. In contrast, when serum 1α,25(OH)_2_D_3_ is in excess, 25(OH)D_3_ is converted into 24,25(OH)_2_D_3_, an inactive form of VD, by CYP24A1 expressing in kidney and other tissues under transcriptional control by 1α,25(OH)_2_D_3_ [8–10]. VD exerts its actions via the nuclear vitamin D receptor (VDR) by direct binding. VDR is a member of the nuclear steroid/thyroid hormone receptor gene superfamily [7]. VDR serves as a ligand-dependent transcriptional activator, and upon ligand binding, VDR binds to target DNA elements to initiate transcription via ligand-dependent associations with various transcriptional co-regulators [11,12]. For stable DNA binding, VDR heterodimerizes with RXRs (RXRα, RXRβ, or RXRγ), which are nuclear vitamin A (retinoid) receptors. The consensus VDR-binding element is composed of two 5′-AGGTCA-3′ motifs separated by 3 bp, and its related elements serve as VD response elements (VDREs) [7,9,13]. As the tertiary structure of the VDR protein is altered by 1α,25(OH)_2_D_3_ binding [14], it is possible to speculate ligand-dependent associations of VDR with co-regulators facilitating gene expression. Numerous co-regulators associate with and regulate nuclear receptors in a ligand binding-dependent manner, and some appear to regulate VDR [8,12,15]. Given the finding that a set of co-regulators modify histone modifications and the chromatin environment via chromatin reorganization [16,17], it is also conceivable that synthetic VDR ligands potently exert tissue-specific biological actions by modulating gene expression and chromatin reorganization regulated by 1α,25(OH)_2_D_3_. This is supported by successful clinical application of synthetic estrogen receptor α (ERα: ERS1) ligands, called selective estrogen receptor modulators (SERMs), as anti-osteoporosis drugs with reduced estrogenic adverse effects [18,19]. Moreover, SERM-bound ERα differs from estrogen-bound ERα in terms of structure and co-regulator recruitment. In this regard, the development of synthetic VDR ligands exerting desirable biological VD activity is promising, as VD is considered safe based on epidemiological evidence and clinical success of long-term treatment. Especially, a synthetic VDR ligand, eldecalcitol (1α,25-dihydroxy-2β-(3-hydroxypropoxy) vitamin D_3_), has been widely applied as an anti-osteoporotic drug in Japan [20,21]. However, at this stage, promising VDR ligands including eldecalcitol are clinically feasible but tend to exert hypercalcemic activity due to their inherent VD activity [22]. To mitigate such undesirable actions of VDR ligands, development of synthetic ligands without calcemic activity but still exerting beneficial biological activity is clearly required.

Thus, the present study was conducted as the initial step in developing a VDR antagonist by generating new synthetic 1α,25(OH)_2_D_3_ derivatives and assessing their gene regulatory actions. VDR antagonists were screened based on the 1α,25(OH)_2_D_3_-induced transactivation function of VDR, and DLAM-**2a**-**d** (DLAM-**2**s) were selected among the synthetic VDR ligands. Using whole genome analyses of cultured human cells treated with VDR ligands, DLAM-**2b** exhibited gene regulatory activity differing from that of 1α,25(OH)_2_D_3_. Thus, we identified a novel VDR ligand with differential gene regulatory activity compared with 1α,25(OH)_2_D_3_.

## Results

### Development and screening of synthetic VDR antagonists

**Fig 1:**
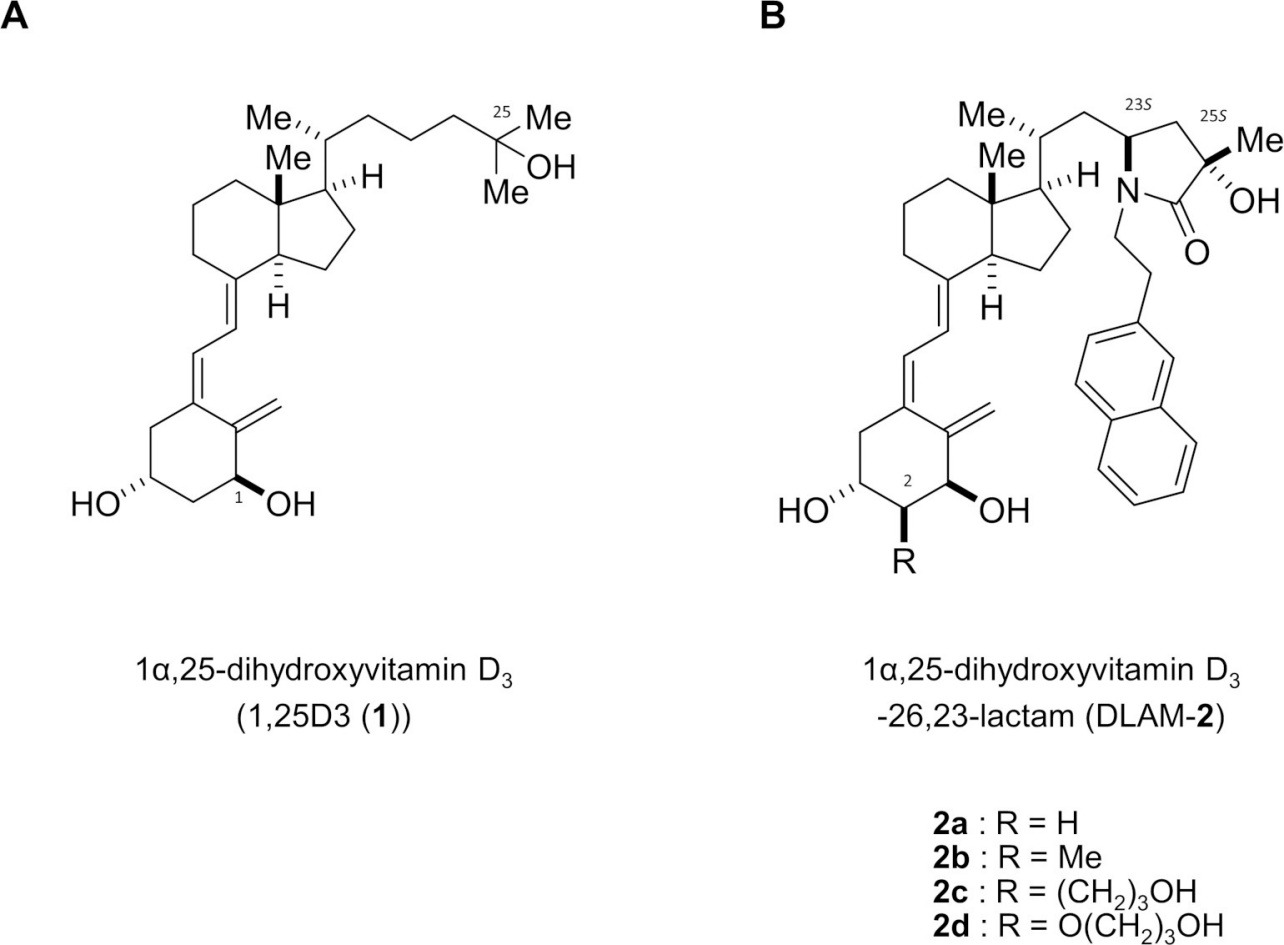
Chemical structures of DLAM derivatives 2a–d. Structures of 1α,25-dihydroxyvitamin D_3_ (1α,25(OH)_2_D_3_ (1) and 1α,25-dihydroxyvitamin D_3_-26,23-lactam (DLAM-2).DOI: 10.6084/m9.figshare.23806737.

VD exerts a variety of beneficial biological actions, and an adequate VD nutritional status is considered to prevent the onset and development of chronic diseases [1–4]. Although synthetic VD analogs have been developed, their calcemic activities have prevented their use in clinical applications [20–22]. To generate VD analogs with the desired biological activity, the present study was conducted as an initial step to identify a VDR antagonist by newly synthesizing synthetic 1α,25(OH)_2_D_3_ derivatives. Among the synthesized compounds, 1α,25(OH)_2_D_3_ derivatives (designated DLAM-**2**s; see their chemical structures in Fig 1 and SI by NMR in S1 Fig) were selected as VDR ligands according to their competitive binding to VDR and antagonistic effect against 1α,25(OH)_2_D_3_ in HOS cells by comparing a known VDR antagonist (TEI-9647) [23] (Table 1).

**Table 1:** Antagonistic property of DLAM-2s in VDR binding and 1α,25(OH)_2_D_3_- induced HL60 cytodifferentiation.

	VD	DLAM-2a	DLAM-2b	DLAM-2c	DLAM-2d
VDR binding affinity (%) ^*a*^	100	19.5	55.6	142.9	111.1
Antagonistic effect (IC_50_, nM) ^*b*^	---	4.49	13.7	6.83	33.4

^*a*^ Potency of 1α,25(OH)_2_ D_3_ for VDR binding affinity is normalized to 100.

^*b*^ The antagonistic activity was assessed in terms of IC_50_ based on osteocalcin gene induction by 10 nM of 1α,25(OH)_2_ D_3_ in an osteogenic cell line derived from human osteosarcoma (HOS cells).

### Chemical synthesis of DLAM derivatives 2a–d

**Fig 2:**
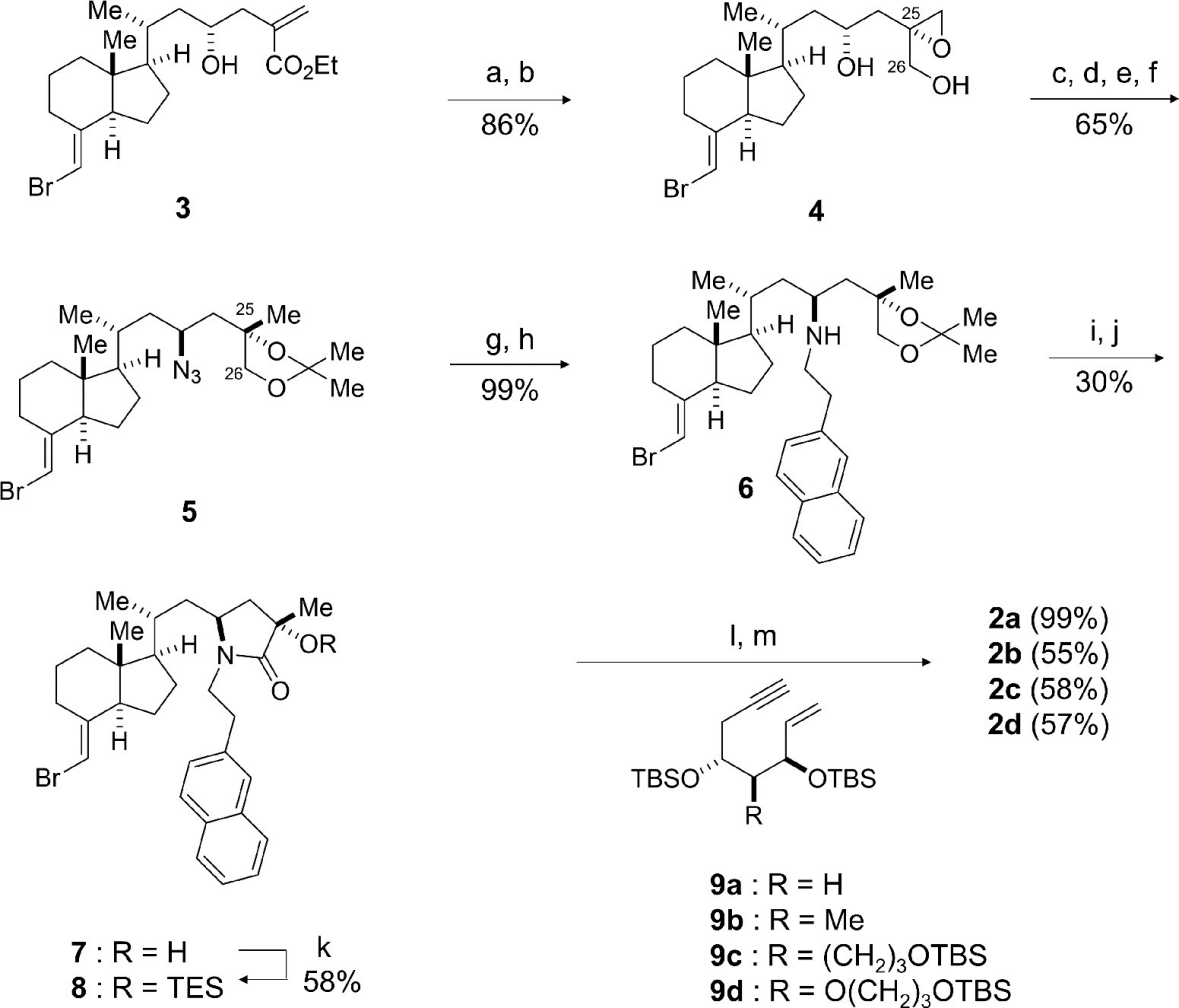
Chemical synthesis of DLAM derivatives 2a–d. Reagents and conditions: (a) DIBAL-H, toluene, 0°C, 1.5 hours; (b) VO (acac)_2_, TBHP, CH_2_Cl_2_, 0°C to room temperature, 30 minutes; (c) LiAlH_4_, THF, 0°C, 30 minutes; (d) p-TsOH・H_2_O, acetone, room temperature, 2 hours; (e) MsCl, Et_3_N, CH_2_Cl_2_, 25°C, 1 hour; (f) NaN_3_, DMF, 60°C, 12 hours; (g) PMe_3_, THF/H_2_O, room temperature to 50°C, 12 hours; (h) 2-(naphthalen-2-yl)acetaldehyde, NaBH_3_CN, MeOH, room temperature, 6 hour; (i) p-TsOH·H_2_O, MeOH, room temperature to 50°C, 9 hours; (j) TEMPO, KBr, NaClO, CH_2_Cl_2_, 0°C, 2 hours; (k) TESOTf, 2,6-lutidine, CH_2_Cl_2_, 0°C, 30 minutes; (l) **9a**–**d**, Pd(PPh_3_)_4_, Et3N, toluene, 90°C, 1 hour; (m) MsOH, MeOH, 0°C to room temperature, 1 hour. DIBAL-H = diisobutylaluminium hydride, acac = acetylacetonate, TBHP = tert-butyl hydroperoxide, THF = tertrahydrofuran, p-TsOH = p-toluenesulfonic acid, MsCl = methylsulfonyl chloride, DMF = N,N-dimethylformamide, TEMPO = 2,2,6,6-tetramethylpiperidine 1-oxyl free radical, TESOTf = triethylsilyl trifluoromethanesulfonate, TES = triethylsilyl, TBS = tert-butyldimethylsilyl, MsOH = methylsulfonic acid. DOI: 10.6084/m9.figshare.23807478. The FID data is available in DOI: 10.6084/m9.figshare.23806101.

Nagasawa et al. developed 1α,25-dihydroxyvitamin D_3_-26-23-lactams (DLAM), bearing a lactam structure in their side chain, as VDR antagonists (Fig 1) [24], and DLAM-2-2-Naphtyl (Nap) (**2a**) was applied as a VDR antagonist in this study. Since the antagonistic activity of DLAM-**2a** was expected to increase by introducing a substituent at C2, derivatives of **2b**–**d** bearing substituents at C2 in **2a** were also synthesized [25,26]. In the synthesis of these DLAM-**2** compounds, a stereoselective synthetic method was newly developed to control the stereochemistry at C23 and C25, which was an issue in the DLAMs synthesized previously [24]. Synthesis of DLAM derivatives **2a–d** is depicted in Fig 2.

Ester **3** [27] was reduced with DIBAL-H to give an allyl/homoallyl alcohol, which was subsequently subjected to Sharpless epoxidation with TBHP as the oxidant in the presence of VO (acac)_2_ as a catalyst to give (23*R*,25*S*)-epoxide **4** predominantly, at a 86% yield and diastereomer ratio of 10:1. After regioselective reduction of the epoxide **4** with lithium aluminum hydride, the resulting 1,2-diol moiety at C25,26 was selectively protected as an acetal with acetone in the presence of *p*-TsOH to give acetonide; the secondary alcohol of acetonide was converted into azide **5** at a 65% yield by reacting with methanesulfonyl chloride in the presence of triethylamine, followed by treatment with sodium azide in DMF at 60°C. After reducing the azide group in **5** under Staudingar reaction conditions with triphenyphosphine and water, the resulting amine was subjected to reductive amination conditions with 2-(naphthalen-2-yl) acetaldehyde and NaBH_3_CN to yield secondary amine **6** with a 2-ethylnaphthalene group, the acetonide group of which was deprotected under acidic conditions to give diol at a 63% yield from **6**. The diol with amine was then subjected to TEMPO and NaClO in the presence of potassium bromide to allow oxidation of the primary alcohol followed by cyclization to lactam **7**, the tertiary hydroxy group of which was protected as triethyl silyl (TES) ether to give the CD ring synthon of **8** at a 58% yield. The DLAM-**2a**-**d** were synthesized by coupling with CD ring **8** and A ring synthons **9a**–**d** [28–30] in the presence of a palladium catalyst followed by deprotection of the triethyl silyl ether group under acidic conditions at 99%, 55%, 58%, and 57% yields, respectively.

### DLAM-2s as VD antagonists in ligand-induced transactivation mediated by VDR in cultured human cells

**Fig 3:**
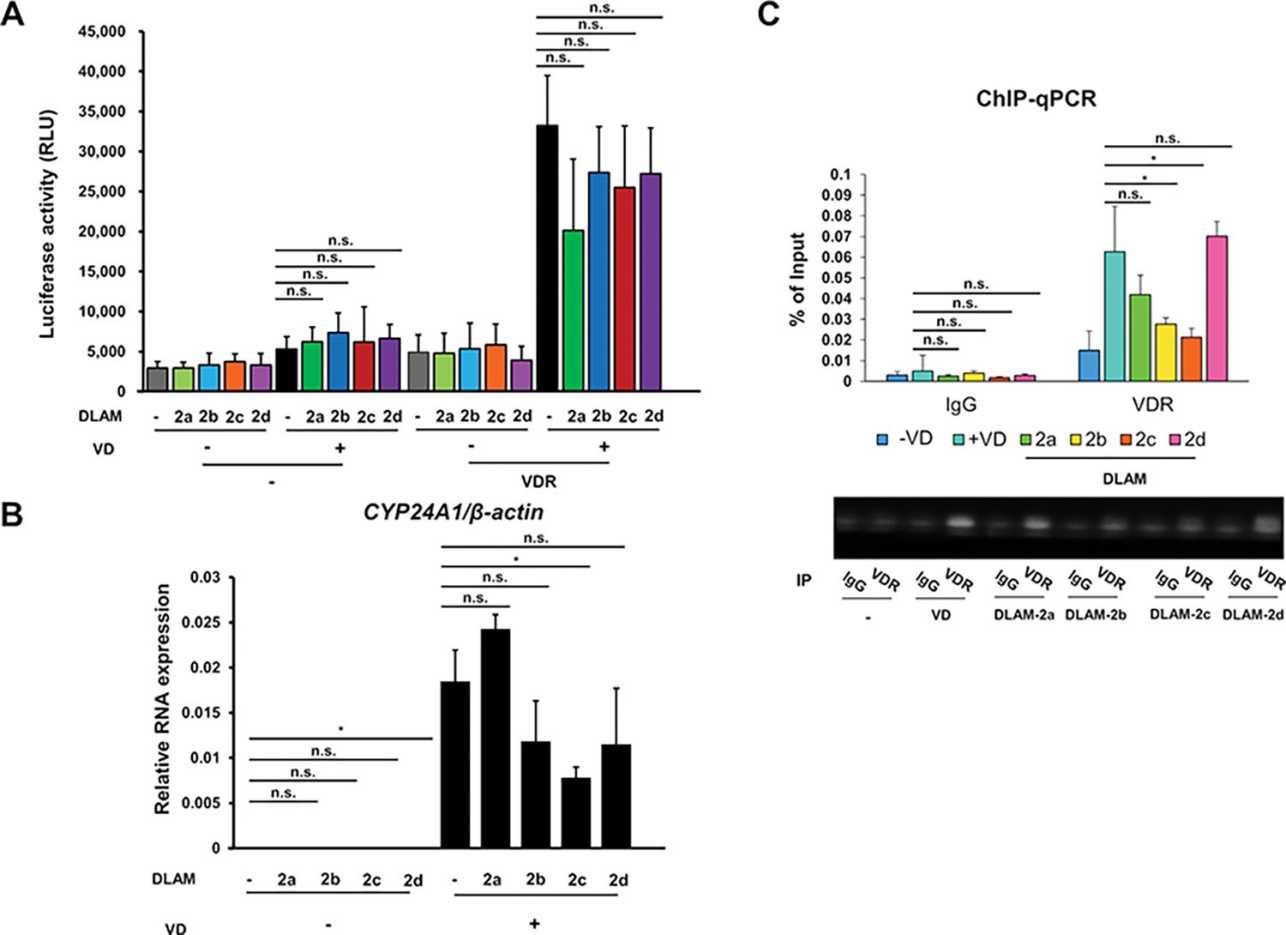
Antagonistic action of DLAM-2s to 1α,25(OH)_2_ vitamin D_3_ for VDR-mediated gene induction in human cultured cells. (A) Luciferase reporter assay using a synthetic VDRE was performed in COS-1 cells treated with 1α,25(OH)_2_D_3_ (depicted as VD in the panel) (100 nM) and/or DLAM-**2**s (1 μM) for 24 h. Values presented are the means ± SD of three independent experiments. (B) Total RNA prepared from HaCaT cells treated with 1α,25(OH)_2_D_3_ (VD) (100 nM) and/or DLAM-**2**s (1 μM) for 4 hours was subjected to quantitative real-time PCR to measure the expression of *CYP24A1*. Values are expressed relative to β-actin and are means ± SD of three independent experiments. (C) ChIP-qPCR analysis of the association of VDR with the CYP24A1 VDRE in HaCaT cells treated with 1α,25(OH)_2_D_3_ (VD)(100 nM) or DLAM-2s (1 μM) for 1.5 hours was performed. Values presented are the means ± SD of three independent experiments. The representative bands of the PCR products were shown by agarose gel electrophoresis. The values in the panels A-C were assessed by post-hoc analysis (Mann-Whitney U-test) after the Kruskal-Wallis test. Statistic differences are shown as “n.s.” for no significance, and “* “for p-value  < 0.05. DOI: 10.6084/m9.figshare.23807481.

To assess if DLAMs act as VD antagonists, their antagonistic activity was assessed in a luciferase assay using an artificial gene reporter plasmid harboring a consensus VDRE in the basal promoter. By transfecting the reporter plasmid with a human VDR expression vector in cultured COS-1 cells in the absence or presence of 1α,25(OH)_2_D_3_, the VD transactivation activity of the DLAM-**2a**–**d** was measured. In the presence of 1α,25(OH)_2_D_3_, the DLAM-**2a**–**d** attenuated the VDR transactivation induced by 1α,25(OH)_2_D_3_ (Fig 3A). DLAM-**2a**–**d** alone had a marginal effect on VDR transactivation. Thus, these findings suggest that DLAMs are, at least partially, synthetic antagonists for VDR.

### DLAM-2b-d attenuation of 1α,25(OH)_2_D_3_-induced expression of endogenous *CYP24A1* in human cultured cells

Based on these findings, DLAM-**2**s were selected among the DLAMs for further study. We evaluated whether DLAM-**2**s act as an antagonist of 1α,25(OH)_2_D_3_ in terms of endogenous gene expression induced by 1α,25(OH)_2_D_3_. *CYP24A1* was evaluated, as it is the most well-characterized target gene of 1α,25(OH)_2_D_3_-bound VDR, with robust induction within a couple of hours of VD exposure [10,31]. Quantitative real-time PCR showed that in HaCaT human keratinocytes, 1α,25(OH)_2_D_3_ treatment effectively induced expression of *CYP24A1*, as expected, and the presence of DLAM-**2b** attenuated the 1α,25(OH)_2_D_3_-induced *CYP24A1* expression (Fig 3B). Treatment with DLAM-**2**s alone hardly induced *CYP24A1* expression, confirming that DLAM-**2a**-**b** act as a 1α,25(OH)_2_D_3_ antagonist on the endogenous expression of the VD target gene.

Since a 1α,25(OH)_2_D_3_ analog acting as a VDR antagonist was recently shown owing to anchoring VDR in the cytosol [32], we next performed a ChIP-qPCR assay in HaCaT cells to assess whether DLAM-**2**s binding to VDR inhibits its binding to target elements in chromatin. A known VDRE located in the human *CYP24A1* promoter was used for VDR recruitment. The presence of 1α,25(OH)_2_D_3_ induced the association of VDR with the *CYP24A1* VDRE, and the addition of DRAM-**2**s did not fully attenuate the VDR association induced by 1α,25(OH)_2_D_3_ (Fig 3C). Thus, these results suggest that VDR bound DLAM-**2**s can recognize and bind to VDREs and related elements in chromatin for gene regulation. As DLAM-**2b**-**d** looked to similarly serve in these assays as VDR ligands, we picked up DLAM-**2b** for further study.

### **No effect of 1**α**,25(OH)**_**2**_**D**_**3**_ **or DLAM-2b on chromatin opening profile between VD and VD antagonist by ATAC-seq analysis**

We recently reported that a clinically applied androgen receptor (AR) antagonist, bicalutamide (Bic), exhibits chromatin remodeling activity, with a gene expression profile different from that induced by an active androgen (dihydrotestosterone [DHT]), in human prostate cancer cells (LNCaP) expressing endogenous AR [33]. Therefore, we evaluated whether the antagonistic effect of DLAM-**2b** on 1α,25(OH)_2_D_3_-induced gene expression mediates chromatin remodeling. Bic antagonized the effect of DHT on AR-mediated gene transactivation and regulation in prostate cancer cell lines. For this purpose, chromatin accessibility was assessed by an ATAC-seq approach in ligand-treated HaCaT cells endogenously expressing VDR in comparison with the effect of Bic in LNCaP cells by calculating chromatin openness in the whole genome. Consistent with our previous report [33], Bic treatment for 5 hours was potent to remodel chromatin (S2A and S2C Fig) with different pattern of DNA-binding motif enrichment with those induced by DHT (S2B Fig). Accordingly, the gene expression profile as well as gene ontology findings was different in the cells treated with DHT and Bic (S3A–S3C Fig). Among the regulated genes, the genes with regulated expression of more than +/- two-fold inductions were picked up (S4A Fig), and only a few (10) of up-regulated genes by AR ligands were overlapped (S4A Fig), suggesting that each of Bic and DHT regulated a different set of the target genes. Among up-regulated genes under co-treatment of DHT with Bic, 160 genes were overlapped, and Bic antagonized expression of 135 genes out of the 160 genes up-regulated by DHT (S4B Fig).

**Fig 4:**
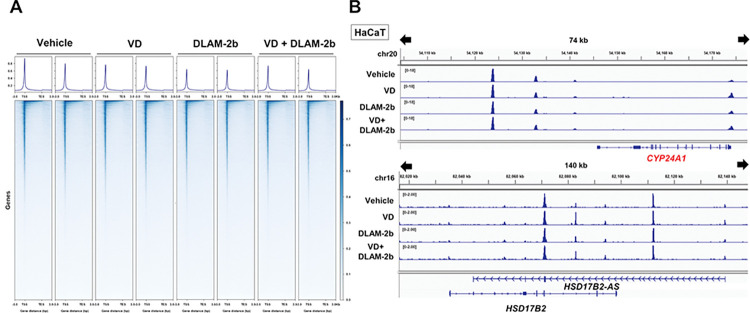
DLAM-2b and 1α,25(OH)_2_ vitamin D_3_ showed no activity to remodel chromatin. Assay for transposases-accessible chromatin (ATAC) analysis was performed in the HaCaT cell line were treated with 1α,25(OH)_2_D_3_ (VD) and/or DLAM-**2b** for 8 hours prior to harvest. (A) Heatmap shows normalized ATAC signals around TSS and TES regions on the whole genome, which visually shows how successful the sequence was. This result indicates different is not significant. (B). Representative sequencing tracks for the gene CYP24A1 and AS-HSD17B2 show ATAC-Seq signals at the promoter and the known enhancer. The data was normalized and the scale on the y-axis was chosen for optimal visualization of peaks for each sample [33]. DOI: 10.6084/m9.figshare.23807487.

**Fig 5:**
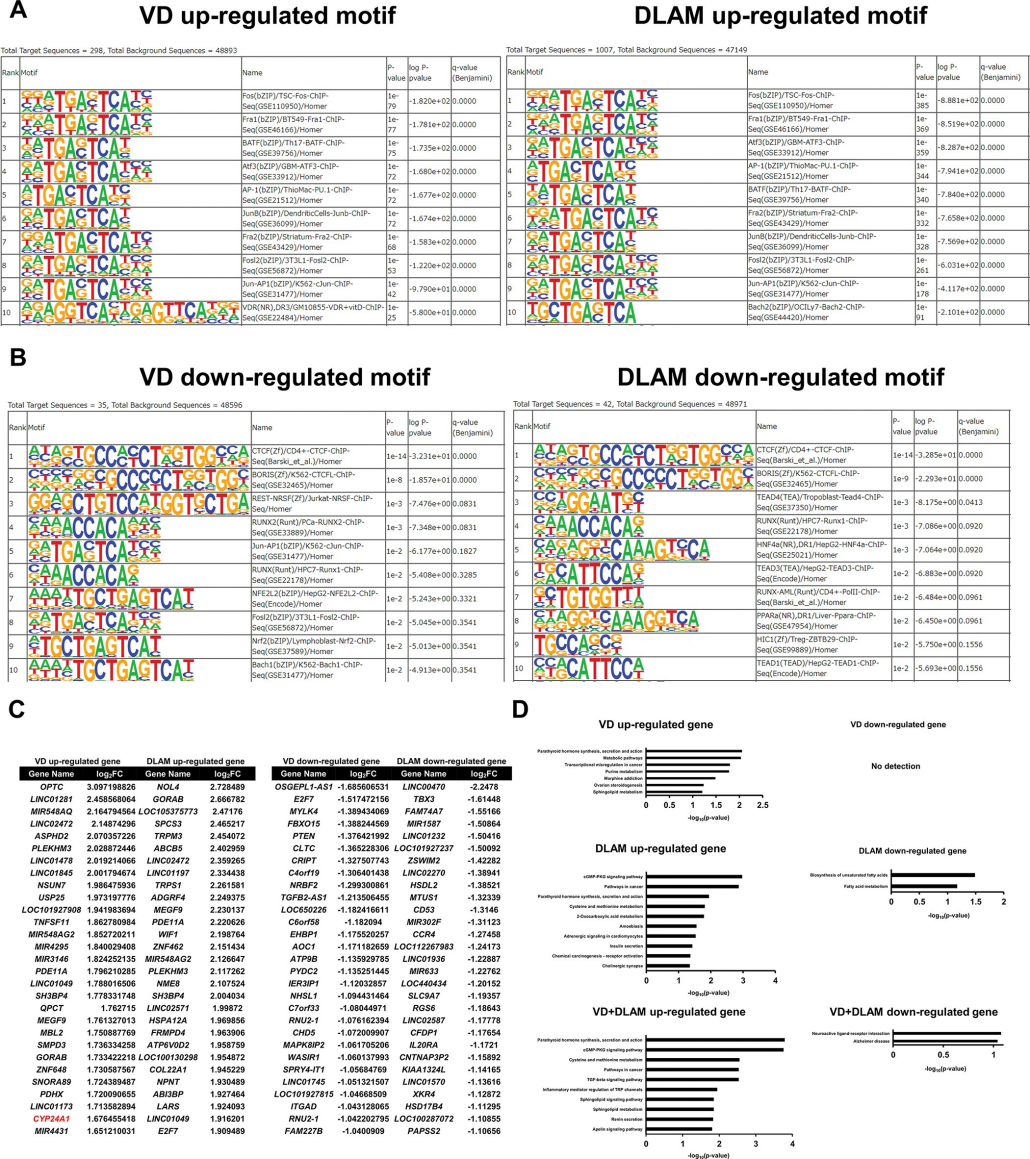
Differential enrichment of transcription factor-binding sequences induced by DLAM-2b and 1α,25(OH)_2_ vitamin D_3_. (A, B) The transcription factor-binding sequences in ATAC-seq peaks based on HOMER (http://homer.ucsd.edu/homer/) analysis in HaCaT cells treated with 1α,25(OH)_2_D_3_(VD) or DLAM-2b for 8 hours were searched and calculated. The top 10 motifs regulated by 1α,25(OH)_2_D_3_ and DLAM-**2b** are shown. (C, D) KEGG pathway analysis of the genes regulated by, 1α,25(OH)_2_D_3_ or DLAM-**2**b. DOI: 10.6084/m9.figshare.23807706.

**Fig 6:**
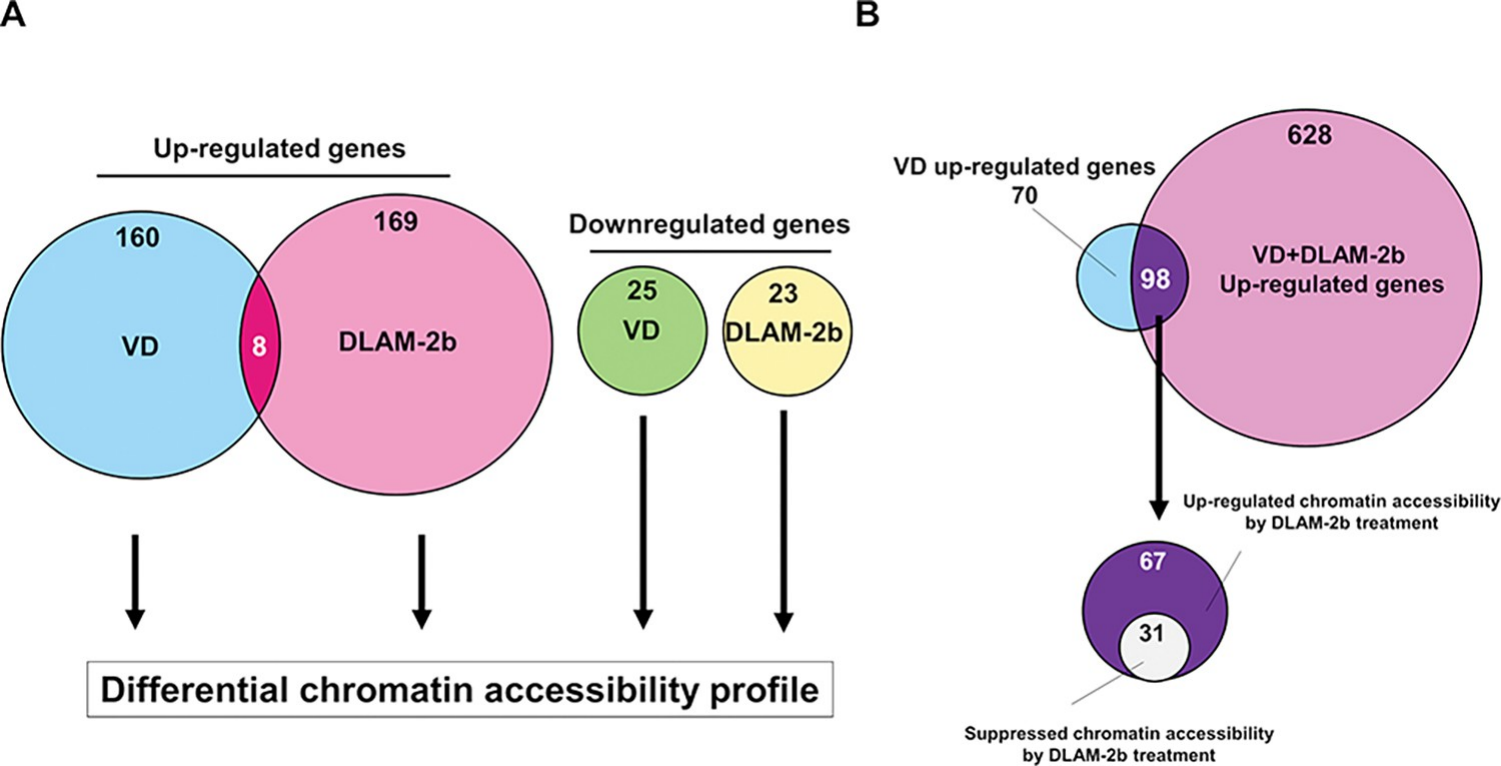
Differential profiles of chromatin accessibility by DLAM-2b and 1α,25(OH)_2_ vitamin D_3_. (A) Venn diagram was used to assess chromatin accessibility profiles of VDR ligand-regulated target regions measured by ATAC-Seq in HaCaT cells. 1α,25(OH)_2_D_3_ (VD) and DLAM-**2b** regulated target regions were selected with p-value<0.05 compared to EtOH that had more than two-fold expression variations were extracted. (B) Venn diagram was used to detect up- or down-regulated effect of DLAM-**2**b for expression of the genes up-regulated by 1α,25(OH)_2_D_3_(VD). DOI: 10.6084/m9.figshare.23807712.

Unlike AR ligands, neither 1α,25(OH)_2_D_3_ nor DLAM-**2b** appeared to remodel the chromatin environment in HaCaT cells, according to the same calculation based on ATAC-seq results (Fig 4A). Consistently, no obvious chromatin reorganization was seen in the loci of the VD target *CYP24A1* and *HSD17B2-AS1* (lncRNA) genes (Fig 4B). Consistently, enrichment profiles of DNA-binding motifs were similar in cells treated with 1α,25(OH)_2_D_3_ versus DLAM-**2b** (Fig 5A and 5B). To assess gene expression profile, the signal peaks of the mRNA coding regions by ATAC-seq were calculated in cells the treated with 1α,25(OH)_2_D_3_ and DLAM-**2b**. The profiles of the regulated gene expression and gene ontology (GO) analysis were different in the cells treated with1α,25(OH)_2_D_3_ versus DLAM-**2b** (Fig 5C and 5D). When closely comparing the regulated genes of more than +/- two-fold inductions out of the regulated genes by Venn diagram, only 8 genes were overlapped among the up-regulated genes by 1α,25(OH)_2_D_3_ (168) and DLAM-**2b** (177) (Fig 6A), while no overlap was seen for the down-regulated genes (Fig 6A). When DLAM-**2b** was co-treated with 1α,25(OH)_2_D_3_, DLAM-**2b** modulated (positively and negatively) expressions of 1α,25(OH)_2_D_3_-regulated genes (Fig 6B), suggesting a partial agonistic activity of DLAM-**2b**. Thus, these findings suggest that DLAM-**2b** exerts a gene regulatory effect beyond simply antagonizing 1α,25(OH)_2_D_3_ activity.

### DLAM-2b as a partial antagonist of 1α,25(OH)_2_D_3_ gene regulation

**Fig 7:**
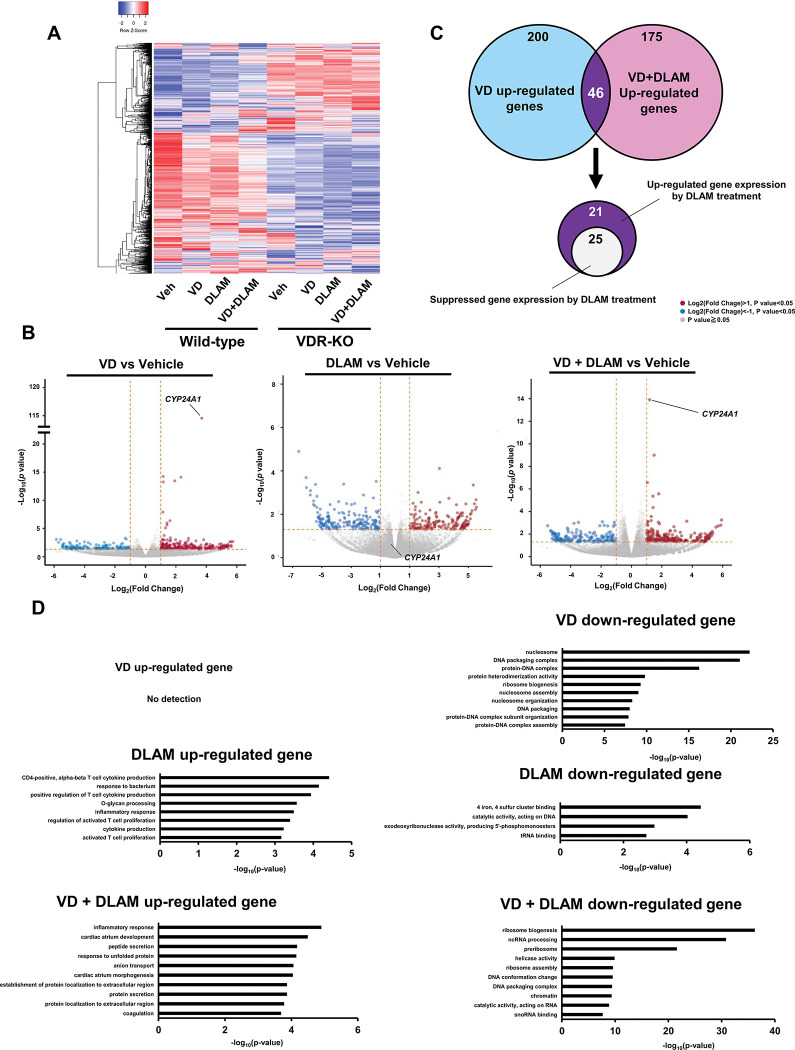
Transcriptome analysis of DLAM-2b action in HCT116 cells. (A) heatmap was created based on the top 2,500 total RNA expression of 4 groups in wild-type and VDR-KO HCT116 cells. The top 2,500 genes were selected by the FPKM data of gene expression. The Heatmapper was used to assess the variation of gene expression by 1α,25(OH)_2_D_3_ (VD) and DLAM-**2b** in the RNA-seq samples (http://www.heatmapper.ca/expression/). Z-score normalization were performed on the normalized read counts across samples for each gene. Z-scores are computed on a gene-by-gene (row-by-row) basis by subtracting the mean and then dividing by the standard deviation. Genes with red were up-regulated and blue were down-regulated. Since the rows were Z-Score scaled, the colors represent a single gene’s varying expression across the samples. The x-axis is the average of three samples. The y-axis is the minus log10 scale of the p values, which indicates the significant level of expression difference. (B) Volcano map of DEGs with treatment of 1α,25(OH)_2_D_3_ (VD) and/or DLAM-**2b** in wild-type HCT116 cells. The ggVolcanoR was used to identify DEGs between 1α,25(OH)_2_D_3_ and/or DLAM-**2b** based on RNA-Seq data (https://ggvolcanor.erc.monash.edu/). The x-axis is the log2 scale of the fold change of gene expression. The y-axis is the minus log10 scale of the p values, which indicates the significant level of expression difference. (C) Co-treatment of DLAM-**2b** with 1α,25(OH)_2_D_3_ was partially suppressive of gene induction by 1α,25(OH)_2_D_3_. (D) GO enrichment analysis was performed to classify the differential genes regulated by 1α,25(OH)_2_D_3_(VD) or DLAM-**2**b. DOI: 10.6084/m9.figshare.23807718.

To evaluate the gene regulatory effect of DLAM-**2b**, we directly compared gene expression profiles in cells treated with 1α,25(OH)_2_D_3_ and/or DLAM-**2b** in HCT116 cell. RNA-seq transcriptome analysis was performed in cells treated with VDR ligands (Fig 7A). Among the regulated genes, the genes of more than +/- two-fold inductions were selected and volcano plot was generated to illustrate gene regulation (Fig 7B). In this plot, the spot representing *CYP24A1* in wild-type cells treated with 1α,25(OH)_2_D_3_ supported the robust induction of *CYP24A1* by 1α,25(OH)_2_D_3_ as well as the antagonistic action of DLAM-**2b** for *CYP24A1* induction 1α,25(OH)_2_D_3_ by (Fig 7B), shown in Fig 3B. While DLAM-**2b** alone appeared not effective *CYP24A1* induction (middle panel of Fig 7B). Among the 246 genes up-regulated by 1α,25(OH)_2_D_3_, DLAM-**2b** co-treatment modulated expressions of only 46 genes, and attenuated the up-regulated expression of the 25 genes (Fig 7C). However, most of the genes (175) regulated by 1α,25(OH)_2_D_3_ + DLAM-**2b** were not overlapped with the genes (200) regulated by 1α,25(OH)_2_D_3_, suggesting a different gene regulatory action of DLAM-**2b** from that by 1α,25(OH)_2_D_3_ (Fig 7C). We then assessed if different gene expression profiles reflect biological output by gene ontology (GO) analysis of the treated cells. Consistent with the GO findings by ATAC-seq, cellular events appeared not fully overlapped (Fig 7D). Thus, these findings are supportive again for a different gene regulatory action of DLAM-**2b** as a VDR ligand from that by 1α,25(OH)_2_D_3_.

### Differential gene expression profiles in cells treated with 1α,25(OH)_2_D_3_ versus DLAM-2b

**Fig 8:**
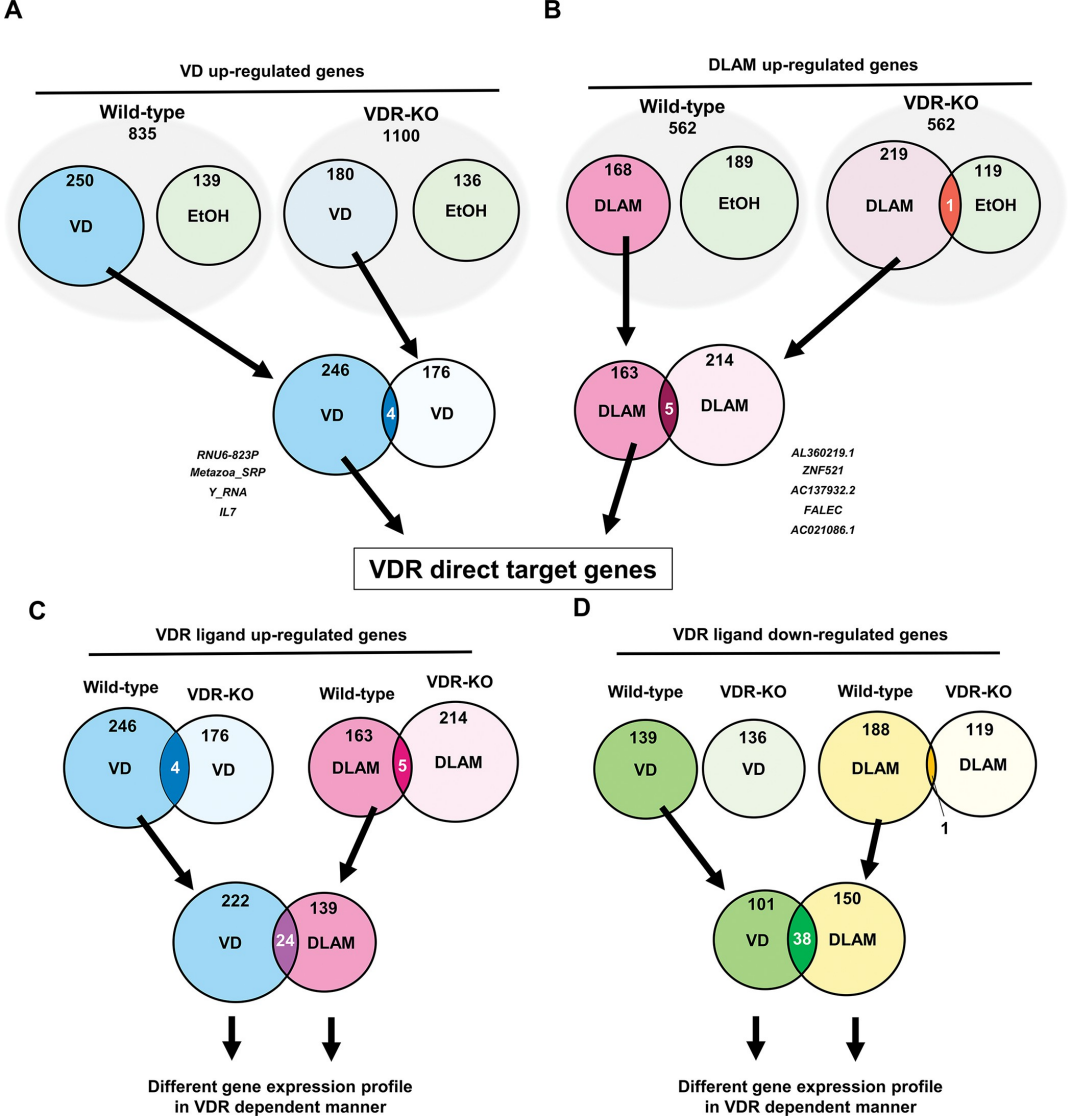
Differential expression profiles of the genes regulated by DLAM-2b and 1α,25(OH)_2_ vitamin D_3_. (A, B) Venn diagram was used to identify VDR target genes regulated by DLAM-**2b** and 1α,25(OH)_2_D_3_ (VD) in wild-type and VDR KO HCT116. The numbers of the VDR target genes regulated by the two VDR ligands are depicted. (C, D) Venn diagram shows differential profiles of VDR target genes regulated by DLAM-**2b** and 1α,25(OH)_2_D_3_. DOI: 10.6084/m9.figshare.23807727.

As synthetic NR ligands exhibit often biological actions in a NR-dependent manner, we applied VDR-knockout (KO) HCT116 cell to verify if DLAM-**2b** indeed mediates VDR as a ligand in the gene regulatory action [10,31]. Four genes showed up-regulation by 1α,25(OH)_2_D_3_ in both of wild-type and VDR-KO cells, so that the remained 246 genes regulated by 1α,25(OH)_2_D_3_ were considered as the VDR target genes (Fig 8A). Likewise, we considered the 163 genes up-regulated by DLAM-**2b** as the VDR targets (Fig 8B). When compared the VDR targets for 1α,25(OH)_2_D_3_ (246) and DLAM-**2b** (163), only 24 genes were overlapped (Fig 8C). For down-regulated genes, similarly only 38 genes were overlapped (Fig 8D). Thus, these findings are supportive again for a different gene regulatory action of DLAM-**2b** as a VDR ligand from that by 1α,25(OH)_2_D_3_.

## Discussion

As 1α,25(OH)_2_D_3_ exerts a wide variety of functions under physiological and pathological settings [1–4], development of synthetic VDR ligands with desirable VD activity is beneficial. All VDR agonists developed so far possess hypercalcemic activity, and hence their clinical application to mitigate pathological events is limited in administrated doses of VDR antagonists [20–22]; this is insufficient to fully achieve the efficiency needed for disease treatment. Therefore, a VDR ligand with no or marginal calcemic activity is ideal for application as a drug with anti-tumor and/or anti-inflammatory activity. While it is still unclear whether the anti-tumor or anti-inflammatory activity of 1α,25(OH)_2_D_3_ is attributed to the transactivation (agonistic) or transrepressive (antagonistic) function of ligand-activated VDR [34–36], the 1α,25(OH)_2_D_3_ calcemic activity is attributed to the ligand-induced transactivation function of VDR [20–22]. The present study was thus conducted to develop a partial VDR antagonist with agonistic activity that retains the beneficial activities, but without the calcemic activity, of 1α,25(OH)_2_D_3_. As a result, four candidates synthetic VDR ligands with antagonistic effects on VDR-mediated transactivation were selected from numerous 1α,25(OH)_2_D_3_ analogues.

In a luciferase reporter assay conducted in cultured cells, DLAMs expectedly antagonized, but only partially, 1α,25(OH)_2_D_3_-induced VDR transactivation and they alone showed a marginal effect on VDR-mediated transactivation in COS-1 cells. Consistently, when compared with 1α,25(OH)_2_D_3_-induced expression of endogenous *CYP24A1* in HaCaT cells, DLAM-**2**s alone had no effect on *CYP24A1* induction yet inhibited 1α,25(OH)_2_D_3_-induced *CYP24A1* expression (Fig 3B). Though TEI-9647 was previously developed as a VDR antagonist based on the VDR transactivation function [23], its antagonistic action for regulation of endogenous gene expression remains elusive. Given the relevant affinity of DLAM-**2**s to VDR compared with that of 1α,25(OH)_2_D_3_ (Table 1), DLAM-**2**s are assumed as partial VDR antagonists. Since the androgen receptor antagonist Bic differentially remodeled the chromatin environment compared with DHT in our recent study [33], we evaluated whether DLAM-**2b** exhibits potent chromatin remodeling activity. Using ATAC-seq in HaCaT cells to assess chromatin openness, unlike AR ligands, neither DLAM-**2b** nor 1α,25(OH)_2_D_3_ affected dynamic chromatin remodeling. However, the gene expression profiles obtained by ATAC-seq and RNA-seq in cells treated with VDR ligands showed that DLAM-**2b** regulated a partially overlapping, but different, set of genes from that regulated by 1α,25(OH)_2_D_3_. These findings suggest that DLAM-**2b** partially antagonizes the effect of 1α,25(OH)_2_D_3_ on gene regulation. The expression profile of the endogenous genes regulated by DLAM-**2b** implies that DLAM-**2b** serves as a VDR partial agonist/antagonist. Though we assume the DLAM-**2b** is a VDR ligand, but cannot exclude a possibility that a subgroup of the genes regulated by DLAM-**2b** is not under targets for VDR bound DLAM-**2b**, but may be due to an off-target effect of DLAM-**2b** through unknown cellular signaling(s). From the gene ontology analysis of the regulated genes by DLAM-**2b** (Figs 5D and 7D), several cellular signaling and cellular events appeared to be modulated by DLAM-**2b,** partially different from 1α,25(OH)_2_D_3_. It is not possible to exclude a possibility at this stage that a part of the modulated events by DLAM-**2b** is owing to off-target action not mediating VDR, since in the VDR KO cells, expressions of a group of the genes were affected (Fig 8). To assess if DLAM-**2b** exerts the assumed tissue-specific action, animal experiment is thus clearly required, from recent reports of synthetic VDR ligands in gene regulation [32,37]. Even though, the findings from the GO analyses by the two approaches are supportive for our idea that DLAM-**2b** behaves like a VDR ligand in gene regulation, leading to a biological outcome, that is different from that by 1α,25(OH)_2_D_3_.

The molecular basis of the gene regulatory effect of DLAM-**2b** remains elusive, but we can speculate that DLAM-**2b** and 1α,25(OH)_2_D_3_ differentially alter the VDR protein structure upon VDR binding. Ligand-induced structural alterations in nuclear receptors are coupled with ligand-dependent associations of transcriptional co-regulators [12,38]. As VDR associates with a number of co-regulators in a 1α,25(OH)_2_D_3_-dependent manner [15], it is conceivable that a specific VDR structure induced by DLAM-**2b** binding affects the ligand binding-induced associations of VDR co-regulators with VDR. Ligand type-specific combinations of VDR and co-regulators might regulate different sets of VDR target genes. This possibility has already been verified with SERMs [18,19]. SERMs are synthetic ERα ligands that act as partial agonist/antagonists of ERα by modulating its N-terminal and C-terminal activation function domains (AF-1 and AF-2, respectively). Since SERMs reportedly activate the AF-1, while repressing the AF-2, function of ERα, SERMs act as agonists in tissues such as bone, where the AF-1 is dominant to the AF-2 in ERα homodimers [39]. The antagonistic actions of SERMs are attributed to suppression of AF-2 function in the female reproductive organs of experimental rodents and humans [18,19,38]. The molecular basis of such tissue-specific actions of SERMs may be SERM-specific alteration of ERα protein structure, following SERM-specific recruitment of co-regulators facilitating the function of the ERα AF-1 and AF-2 [38,40]. Unlike ERα, VDR contains only the AF-2, as the A/B domain of VDR is too short to serve as an AF-1 domain for docking of co-regulators [8,13]. However, as VDR heterodimerizes with RXRα, RXRβ, or RXRγ, the AF-1 function of the RXR might compensate for the lack of VDR AF-1 function in the heterodimer [41].

Regarding the structure of VDR altered by DLAM-**2b** binding compared with 1α,25(OH)_2_D_3_ binding, Rovito *et al*. recently illustrated ligand-type alterations in the ligand-binding domain (LBD) of VDR [32]. By comparing shifts in the angle of the VDR LBD helix induced by 1α,25(OH)_2_D_3_, the VDR ligand ZK168281 effectively altered the VDR LBD. Consequently, nuclear localization of VDR-bound ZK168281 was impaired, partially accounting for the antagonistic activity of this compound [32]. Gene expressed profiles among the five known VDR targets determined by RT-qPCR assay revealed 1α,25(OH)_2_D_3_ agonistic and antagonist activities of ZK168281. More strikingly, this compound ameliorated the hypercalcemia induced by excess 1α,25(OH)_2_D_3_ in intact mice. Considering the findings of Rovito *et al*. and our group, the agonistic/antagonist action of DLAM-**2b** in gene regulation could be due to the differentially altered structure of VDR, and this hypothesis remains to be tested at the protein structure level.

Differential effects of SERMs in association with ERα co-regulators other than estrogen have been described, but only a limited number of co-regulators among the known ERα co-regulators have been evaluated at this stage [12,38]. In addition to histone modifiers and chromatin-remodeling factors regulating ERα, a pioneer factor (FOXA1) regulates the effects of ERα on chromatin by advanced DNA binding of FOXA1 to target elements near ERα-binding sites, in prior to facilitating DNA binding of ERα [42]. Although FOXA1 has a similar effect on AR, no such pioneer factors for VDR are known. Seuter *et al*. reported in THP-1 human monocytic leukemia cells that PU.1 is a pioneer factor for VDR [43], but the role of PU.1 in other cell lines and intact animals remains to be addressed. As mice deficient in PU.1-related genes do not have impaired VDR function or VD signaling in VD target tissues, the PU.1 function as a VDR pioneer factor might be limited to only a few cell types. In support of this, clear effects of 1α,25(OH)_2_D_3_ and DLAM-**2b** on chromatin reorganization were not detected in tested cell lines endogenously expressing VDR (Fig 4). Even if a VDR pioneer factor is absent in cell lines, we provide evidence from gene expression profiles using ATAC-seq and RNA-seq approaches that VDR is a cryptic modulator of gene regulation controlled by ligands. In this respect, it would be interesting to determine whether known VDR co-regulators associate with VDR-bound DLAM-**2b**. Furthermore, VDR-bound DLAM-**2b** may act as a biochemical bait for screening uncharacterized co-regulators.

In conclusion, in this study, we found that DLAM-**2b** is a VD partial antagonist/agonist in gene regulation in human cultured cells. However, to verify this idea, animal experiment to assess in vivo action of DLAM-**2b** is clearly required. Also, the present findings imply the significance of genome wide analysis in development of a novel type of synthetic VDR ligand.

## Materials and methods

### VDR binding assay

[26,27-methyl-3H]-1α,25-dihydroxyvitamin D_3_ (specific activity 6.623 TBq/mmol, 15,000 dpm, 15.7 pg) and various amounts of 1α,25(OH)_2_D_3_ and an analog to be tested were dissolved in 50 μl absolute ethanol in 12 × 75 mm polypropylene tubes. Then, 0.2 mg chick intestinal VDR and 1 mg gelatin in 1 ml phosphate buffer solution (25 nM KH2PO4, 0.1 M KCl, 1 mM dithiothreitol, pH 7.4) were added to each tube in an ice bath. The assay tubes were incubated in a shaking water bath for 1 hour at 25°C and then chilled in an ice bath, after which 1 ml 40% polypropylene glycol 6000 in distilled water was added to each tube. The tubes were mixed vigorously and centrifuged at 2260 × g for 60 minutes at 4°C. After the supernatant was decanted, the bottom of the tube containing the pellet was cut off into a scintillation vial containing 10 ml dioxane-based scintillation fluid, and the radioactivity was measured using a Beckman liquid scintillation counter (Model LS6500). The relative potency of the analogs was calculated from the concentration of the analog needed to displace 50% of [26,27-methyl-3H]-1α,25-dihydroxyvitamin D_3_ from the receptor compared with the activity of 1α,25(OH)_2_D_3_ (final concentration: 10^8^ M) (assigned a 100% value).

### Cell culture and in vitro experiments

The human cell lines (HCT116, COS-1, HaCaT and LNCaP) were provided by the RIKEN BRC. The cells were cultured in Dulbecco’s Modified Eagle’s Medium, low glucose (Nacalai, Kyoto, Japan), supplemented with 10% heat-inactivated FCS (Biological Industries, Beit HaEmek, Israel). VDR-KO HCT116 cells were generated using a versatile non-homologous end joining-based knock-in module for genome editing (VIKING) method [10,44]. Cell culture was performed at 37°C under 5% CO_2_. For RNA-seq, 1.0 × 10^6^ cells were seeded in a 6-well plate and stimulated with 1α,25(OH)_2_D_3_ (final concentration: 100 nM) and/or DLAM-2P-2Naph-2α-nPrOH (**2c**) (final concentration: 1 μM) reagent for 8 hours, after which the cells were harvested for RNA extraction.

### Luciferase assay

#### Screening of synthetic VDR ligands

Plasmids of an VDR expression vector [pGL4.26 DR3 pcDNA3-human VDR (Full length)] and a luciferase reporter gene [pGL4-CMV-Rluc (Promega)] (ratio = 5:1) were transfected in HOS cells (purchased from ATCC) by using MaxCyte STX (MaxCyte Co. Ltd), and the transfected cells after incubation at 37°C under 5% CO2 for 20 h were cryopreserved. The frozen transfected cells were thawed and suspended into DMEM media containing 5% charcoal stripped Fatal Bovine Serum. Transfected cells were seeded onto 384-well plate (4000 cells / 10 μL / well) and incubated under 5% CO2 at 37°C for 4 h. Synthetic compounds were dissolved in 100% DMSO and added to the wells (The final concentration of DMSO in the assay was 0.1%). The DMSO solution of 1α,25(OH)_2_D_3_ (final concentration: 10^8^ M) and an analog (final concentration: 10^11^ to 10^6^ M) was added to the culture medium at 0.1% volume, and after 20 h incubation at 37°C under 5% CO2, the Dual-Glo Luciferase Assay System (Pro-Mega) was used to assess transactivation activity of the tested compounds according to the manufacture’s instructions, and EC50 values were calculated using the XLfit program (ID business Solution Ltd.) [23].

#### Evaluation of antagonistic activity of DLAM-2 compounds

For transfection, cells were grown to 50% confluency in 12-well plates. Transfection was performed using Lipofectamine^TM^ 3000 Transfection Reagent (Thermo Fisher Scientific, Carlsbad, CA, USA) according to the manufacturer’s protocol. The total amount of DNA used for transfection was adjusted by adding empty vector. Luciferase activity was measured using the Dual Luciferase Reporter Assay System (Promega, Madison, WI, USA). The reporter plasmid containing luciferase reporter gene *luc2* (pGL4.27[luc2P/minP/Hygro]) and a human VDRE in the promoter, together with a reference Renilla luciferase plasmid was used to adjust for differences in transfection efficiency [10,33].

All values are reported as means ± SE from at least three independent experiments. The Kruskal–Wallis tests were performed to compare the ligand action and Mann-Whitney U-test were performed for post hoc test.

#### Quantitative real-time RT-PCR

Total RNA extracted from the cells was prepared using Trizol regent (Thermo Fisher Scientific, Carlsbad, CA, USA) according to the manufacturer’s protocol. cDNA was synthesized using ReverTra Ace® qPCR RT Master Mix with gDNA Remover (TOYOBO, Osaka, Japan). Quantitative real-time RT-PCR was conducted using SYBR Green Master mix on the CFX Connect Real-Time PCR System (Bio-Rad, Hercules, CA, USA). All relative RNA expression values were calculated using the 2^−ΔΔCt^ method with normalization to β-actin expression [10,31]. The Kruskal–Wallis tests were performed to compare the ligand action on the *CYP24A1* expression and Mann-Whitney U-test were performed for post hoc test.

### ChIP-qPCR assay

ChIP was performed using the ChIP-IT Express Kit (Active Motif, Carlsbad, CA, USA) with anti-VDR (rabbit, 1:40; Cell Signaling Technology, Danvers, MA, USA, #12550) and rabbit IgG (mouse, 1:400; Cell Signaling Technology, Danvers, MA, USA, #3900) antibodies[31]. For qPCR, ChIP samples were subjected to DNA clean up using the NucleoSpin Gel and PCR Clean-up kit (Macherey Nagel, Düren, Germany) according to the manufacturer’s protocol. qPCR was conducted using iTaq^TM^ Universal SYBR Green Supermix (Bio-Rad, Hercules, CA, USA). The Kruskal–Wallis tests were performed to compare the effect on VDR recruitment to the VDRE located in the human *CYP24A1* promoter and Mann-Whitney U-test were performed for post hoc test.

### RNA-seq

RNA-seq library preparation, sequencing, mapping, gene expression analysis, and gene ontology (GO) enrichment analysis were performed by DNAFORM (Yokohama, Kanagawa, Japan). Total RNA quality was confirmed using a Bioanalyzer (Agilent) to ensure that the RNA integrity number was greater than 7.0. After poly(A) RNA enrichment using the NEBNext Poly(A) mRNA Magnetic Isolation Module (New England BioLabs, Ipswich, MA, USA), double-stranded cDNA libraries (RNA-seq libraries) were prepared using the SMARTer Stranded Total RNA Sample Prep Kit–HI Mammalian (Clontech, Mountain View, CA, USA) according to the manufacturer’s instruction. RNA-seq libraries were sequenced using paired-end reads (50 nucleotides of read 1 and 25 nucleotides of read 2) on the NextSeq 500 instrument (Illumina, San Diego, CA, USA). The obtained raw reads were trimmed and quality-filtered using Trim Galore! (ver. 0.4.4), Trimmomatic (ver. 0.36), and cutadapt (ver. 1.16) software. The trimmed reads were mapped to the human GRCh38 genome using STAR (ver. 2.7.2b). Reads of annotated genes were counted using featureCounts (ver. 1.6.1). Annotation of gene information to the peaks was performed by default settings of the software HOMER based on information in the database RefSeq. FPKM values were calculated from mapped reads after normalizing to the total counts and transcript length. Differentially expressed genes were detected using the DESeq2 package (ver. 1.20.0). Gene information were based on the public database Ensembl. The differentially expressed genes detected by DESeq2 (base mean > 5 and fold-change expression < 0.25, or base mean > 5 and fold-change expression > 4) were subjected to GO enrichment analysis and Kyoto Encyclopedia of Genes and Genomes (KEGG) pathway analysis. GO enrichment analysis were performed by the clusterProfiler package [45]. Peak’s outlier determination was performed using Cook’s distance. KEGG pathway analysis were performed by Database for Annotation, Visualization, and Integrated Discovery (DAVID) online software [46].

### ATAC-seq

HaCaT and LNCaP cells were seeded in 100 mm dishes in duplicate, and the culture medium was exchanged with FCS-free medium after 24 hours. At 80–90% confluency, treatment with 1α,25(OH)_2_D_3_ (final concentration: 100 nM) and/or DLAM-2b (final concentration: 1 μM) for 8 hours was performed after renewing the medium again. Cells were harvested using 0.1% trypsin and 0.02 EDTA, and the cells were counted to determine cell viability because over 1.0 x 10^5^ viable cells are needed for ATAC-seq. The harvested viable cells (vehicle group: 6.2 x 10^5^ and 2.5 x 10^5^; VD group: 2.8 x 10^5^ and 5.4 x 10^5^; DLAM-2b group: 6.9 x 10^5^ and 8.2 x 10^5^; and VD plus DLAM-2b group: 3.2 x 10^5^ and 4.2 x 10^5^), respectively, were preserved using CELLBANKER^®^1 plus and submitted for ATAC-seq.

ATAC-seq library preparation, sequencing, mapping, gene expression analysis, and gene ontology (GO) enrichment analysis were performed by DNAFORM (Yokohama, Kanagawa, Japan). Fragmentation and amplification of the ATAC-seq libraries were conducted according to a previous method [47]. Briefly, approximately 50,000 cells were lysed and the lysate subjected to a transposition reaction using Tn5 transposase (Illumina Catalog #FC121-1030) at 37°C for 30 minutes. The reaction liquid was purified using the Qiagen MinElute PCR Purification Kit. Then, five cycles of PCR were conducted using custom Nextera PCR primers and the NEBNext Q5 Hot Start HiFi PCR Master Mix (New England Biolabs) [48]. The need for additional PCR cycles was determined by qPCR of the partially amplified products, following the reported protocol [47]. The PCR products were purified using Agencourt AMPure XP beads (Beckman Coulter: A63881) by double size selection (left ratio: 1.4, right ratio: 0.5) according to the manufacturer’s protocol. Paired-end sequencing was performed on the DNBSEQ-G400RS High-throughput Sequencing Set (MGI Tech, Shenzhen, China). Mapping and peak calling were conducted using the ENCODE ATAC-seq pipeline (https://github.com/ENCODE-DCC/atac-seq-pipeline). Reads were mapped to the GRCh38.p13 (hg38) reference sequence using Bowtie2 (ver. 2.3.4.3), and duplicate reads were removed using Picard (ver. 2.20.7) and samtools (ver. 1.9). Peak calling was performed using MACS2 (ver. 2.2.4) with the default parameters. Determination of blacklist regions was performed using Cook’s distance. After removal of blacklist regions, the consistency of the peaks was evaluated by the irreproducible discovery rate using IDR (ver. 2.0.4.2). Peak annotation was conducted using HOMER (ver. 4.9.1) with the default settings. Gene informations were based on the public database Ensembl. The differentially accessible peaks were obtained using DEseq2 (ver. 1.20.0). Known motifs and de novo consensus motifs within the central 200 bp of the obtained peaks were searched using HOMER with the default settings. The known motifs were used motif database (Homer motif database) included in HOMER.

### Synthesis of DLAM 2a-d

#### General experimental methods

Unless otherwise stated, reactions were performed under an argon atmosphere using freshly dried solvents. All reactions were monitored by thin-layer chromatography using Merck silica gel 60 F_254_ pre-coated plates (0.25 mm) and were visualized by UV, *p*-anisaldehyde staining, and Hanessian staining. Flash column chromatography was performed under pressurization using silica gel (particle size 40~100 μm) purchased from Cica (Kanto Chemical Co., Tokyo, Japan). ^1^H spectra were recorded on JNM-ECX 500, JNM-ECX 400 or JMTC 300. The spectra are referenced internally according to residual solvent signals of CDCl_3_ (^1^H-NMR; *δ* = 7.26 ppm). Data for ^1^H NMR spectra are recorded as follows: chemical shift (*δ*, ppm), (multiplicity, coupling constant (Hz), integration). Multiplicity and qualifier abbreviations are as follows: s = singlet, d = doublet, t = triplet, q = quartet, dd = double doublet, m = multiplet, br = broad. The SI data of compounds by NMR are shown in S1A–S1R Fig.

#### (2R,4R)-4-((1R,3aR,7aR,E)-4-(Bromomethylene)-7a-methyloctahydro-1H-inden-1-yl)-1-((S)-2-(hydroxymethyl)oxiran-2-yl)pentan-2-ol (4)

To a suspension of ester **3** (Fig 2) (267.3 mg, 0.647 mmol) in toluene (6.8 mL) was added DIBAL-H (1.0 M in toluene, 2.1 mL, 2.1 mmol) at 0°C under argon, and the reaction mixture was stirred at the same temperature for 1.5 h. After the reaction, to the mixture was added 2-propanol (1.5 mL), H_2_O, and SiO_2_, and the resultant was stirred for 30 min and filtered through a pad of celite. The filtrates were concentrated *in vacuo*, and the residue was purified by column chromatography on silica gel (*n*-hexane/ethyl acetate = 4:1) to give diol (226.0 mg, 94%). To a solution of diol (88.5 mg, 0.24 mmol) in CH_2_Cl_2_ (2.4 mL) was added VO (acac)_2_ (6.3 mg, 0.024 mmol) and TBHP (5.5 M in decane, 0.26 mL, 1.43 mmol) at 0°C under argon, and the reaction mixture was stirred at room temperature for 30 min. To the mixture was added sat. Na_2_S_2_O_3_ aq. (2.4 mL) and the organic layer was extracted with dichloromethane. The organic layer was dried over MgSO_4_, filtered, and concentrated *in vacuo*. The residue was purified by column chromatography on silica gel (*n*-hexane/ethyl acetate = 2:1) to give **4** (85.7 mg, 92%).

Spectral data for **4**: [α]^25^_D_ = +38.1 (*c* 0.3, CHCl_3_); ^1^H NMR (300 MHz, CDCl_3_) *δ* 5.65 (s, 1H), 4.00 (t, *J* = 9.5 Hz, 1H), 3.76 (m, 2H), 2.87 (d, *J* = 4.5 Hz, 1H), 2.73 (d, *J* = 4.5 Hz, 1H), 2.08–1.6 (m, 10H), 1.40–1.27 (m, 7H), 1.01 (d, *J* = 6.6 Hz, 3H), 0.59 (s, 3H) ppm; ^13^C NMR (75 MHz, CDCl_3_) *δ* 144.9, 97.5, 66.3, 64.6, 58.9, 56.2, 55.8, 50.8, 45.5, 44.3, 41.2, 39.8, 32.5, 30.9, 27.7, 22.5, 22.0, 18.6, 11.9 ppm; HRMS: Calcd. for C_19_H_31_BrNaO_3_ 409.1354, found 409.1332.

#### (S)-4-((2S,4R)-2-Azido-4-((1R,3aR,7aR,E)-4-(bromomethylene)-7a-methyloctahydro-1H-inden-1-yl)pentyl)-2,2,4-trimethyl-1,3-dioxolane (5)

To a solution of **4** (85.7 mg, 0.22 mmol) in THF (2.2 mL) was added LiAlH_4_ (37.9 mg, 1.00 mmol) at 0°C under argon, and the reaction mixture was stirred at the same temperature for 1 h. To the mixture was added H_2_O (37.9 μL), 15% NaOH aq. (37.9 μL) and H_2_O (113.7 μL), and the resultant was stirred for 15 min. Then the mixture was dried over MgSO_4_, stirred for 15 min, and filtered through a pad of celite. The filtrates were concentrated *in vacuo*, and the residue was purified by column chromatography on silica gel (*n*-hexane/ethyl acetate = 4:1) to give triol (73.8 mg, 86%). To a solution of triol (118.1 mg, 0.304 mmol) in acetone (3.0 mL) was added *p*-TsOH·H_2_O (2.9 mg, 0.015 mmol) at room temperature, and the reaction mixture was stirred at the same temperature for 2 h. To the mixture was added Et_3_N (0.1 mL) and the resultant was concentrated *in vacuo*. The residue was purified by column chromatography on silica gel (*n*-hexane/ethyl acetate = 20:1) to give alcohol with acetonide (98.6 mg, 76%). To a solution of the alcohol with acetonide (154.1 mg, 0.360 mmol) in CH_2_Cl_2_ (1.9 mL) was added Et_3_N (0.24 mL, 1.74 mmol) and MsCl (0.090 mL, 1.16 mmol) at −25°C under argon, and the reaction mixture was stirred at the same temperature for 1 h. To the mixture was added H_2_O (1.9 mL) and the organic layer was extracted with dichloromethane for three times. The combined organic layer was dried over Na_2_SO_4_, filtered, and concentrated *in vacuo*, and the residue was purified by column chromatography on silica gel (*n*-hexane/ethyl acetate = 8:1) to give methane sulfonate (184.7 mg, >99%). Due to the instability, this compound was used to the next step. To a solution of methane sulfonate (195.9 mg, 0.387 mmol) in DMF (3.9 mL) was added NaN_3_ (75.5 mg, 1.16 mmol) at room temperature under argon, and the reaction mixture was stirred at 60°C for 12 h. To the mixture was added H_2_O (3.9 mL) and the organic layer was extracted with ethyl acetate for three times. The combined organic layer was washed with water and brine, dried over MgSO_4_, filtered, and concentrated *in vacuo*. The residue was purified by column chromatography on silica gel (*n*-hexane/ethyl acetate = 80:1) to give **5** (194.3 mg, >99%).

Spectral data for **5**: [α]^25^_D_ = +51.3 (*c* 0.2, CHCl_3_); ^1^H NMR (300 MHz, CDCl_3_) *δ* 5.64 (s, 1H), 3.98 (d, *J* = 8.3 Hz, 1H), 3.75 (d, *J* = 8.3 Hz, 1H), 3.44 (m, 1H), 2.89 (m, 1H), 1.98 (m, 3H), 2.00–1.30 (m, 13H), 1.42 (s, 3H), 1.33 (s, 3H), 0.99 (d, *J* = 5.9 Hz, 3H), 0.57 (s, 3H) ppm; ^13^C NMR (75 MHz, CDCl_3_) *δ* 144.8, 109.4, 97.6, 79.8, 73.5, 56.6, 56.2, 55.7, 45.5, 42.7, 41.6, 39.8, 33.9, 30.9, 27.8, 27.1, 26.7, 26.1, 22.4, 22.0, 18.9, 11.8 ppm; HRMS: Calcd. for C_22_H_36_BrN_3_NaO_2_ 476.1889, found 476.1876.

#### (2S,4R)-4-((1R,3aR,7aR,E)-4-(Bromomethylene)-7a-methyloctahydro-1H-inden-1-yl)-N-(2-(naphthalen-2-yl)ethyl)-1-((S)-2,2,4-trimethyl-1,3-dioxolan-4-yl)pentan-2-amine (6)

To a solution of **5** (175.4 mg, 0.387 mmol) in THF (3.6 mL) was added PMe_3_ (1.0 M in THF, 1.2 mL, 1.16 mmol) at 0°C under argon, and the reaction mixture was stirred at room temperature for 1.5 h. Then H_2_O (0.36 mL) was added, and the resulting mixture was stirred at 50°C for 12 h. After the reaction, the mixture was concentrated *in vacuo* to give the corresponding amine. The resulting amine and 2-(naphthalen-2-yl) acetaldehyde (131.6 mg, 0.779 mmol) was dissolved in MeOH (3.9 mL) at room temperature under argon, and NaBH_3_CN (26.8 mg, 0.426 mmol) was added to the mixture, and the resulting mixture was stirred at the same temperature for 6 h. After the reaction, the mixture was concentrated *in vacuo*, and the residue was purified by column chromatography on silica gel (*n*-hexane/ethyl acetate = 4:1) to give **6** (269.7 mg, >99%).

Spectral data for **6**: [α]^25^_D_ = +58.5 (*c* 0.4, CHCl_3_); ^1^H NMR (300 MHz, CDCl_3_) *δ* 7.78 (m, 3H), 7.70 (brs, 1H), 7.44 (m, 3H), 5.63 (s, 1H), 3.95 (t, *J* = 6.5 Hz, 1H), 3.70 (d, *J* = 8.1 Hz, 1H), 3.65 (d, *J* = 8.1 Hz, 1H), 3.05 (t, *J* = 6.5 Hz, 2H), 2.86 (m, 3H), 1.97 (m, 2H), 1.69–1.16 (m, 12H), 1.16 (s, 3H), 1.10 (s, 3H), 0.95 (d, *J* = 5.1 Hz, 3H), 0.55 (s, 3H) ppm; ^13^C NMR (100 MHz, CDCl_3_) *δ* 145.0, 133.5, 132.2, 128.0, 127.5, 127.4, 127.1, 127.1, 125.9, 125.2, 109.6, 97.5, 80.9, 75.7, 60.4, 56.4, 55.8, 53.1, 48.2, 45.4, 39.8, 36.4, 33.5, 30.9, 27.9, 26.8, 26.4, 24.4, 22.5, 22.0, 21.0, 19.0, 14.2, 11.9 ppm; HRMS: Calcd. for C_34_H_49_BrNO_2_ 582.2947, found 582.2945.

#### (3S,5S)-5-((R)-2-((1R,3aR,7aR,E)-4-(bromomethylene)-7a-methyloctahydro-1H-inden-1-yl)propyl)-3-hydroxy-3-methyl-1-(2-(naphthalen-2-yl)ethyl)pyrrolidin-2-one (7)

To a solution of **6** (40.4 mg, 0.070 mmol) in MeOH (1.6 mL) was added *p*-TsOH·H_2_O (30 mg, 0.160 mmol) at room temperature under argon, and the reaction mixture was stirred at 50°C for 9 h. To the reaction mixture was added sat. NaHCO_3_ aq. (1.6 mL) and the organic layer was extracted with ethyl acetate for three times. The combined organic layer was washed with brine, dried over MgSO_4_, filtered, and concentrated *in vacuo*. The residue was purified by column chromatography on silica gel (chloroform/methanol = 50:1) to give diol (23.8 mg, 63%). To a solution of the diol (5.7 mg, 0.011 mmol) in CH_2_Cl_2_ (0.053 mL) was added NaClO (0.018 mL, 0.264 mmol), KBr (1.3 mg, 0.011 mmol) and TEMPO (0.7 mg, 0.002 mmol) at 0°C, and the mixture was stirred at the same temperature for 2 h. To the reaction mixture was added H_2_O (0.1 mL) and the organic layer was extracted with dichloromethane for three times. The combined organic layer was dried over MgSO_4_, filtered, and concentrated *in vacuo*, and the residue was purified by column chromatography on silica gel (*n*-hexane/ethyl acetate = 3:1) to give **7** (2.7 mg, 48%).

Spectral data for **7**: [α]^25^_D_ = +120.8 (*c* 0.3, CHCl_3_); ^1^H NMR (300 MHz, CDCl_3_) *δ* 7.78 (m, 3H), 7.64 (brs, 1H), 7.44 (m, 2H), 7.35 (dd, *J* = 9.5, 2.5 Hz, 1H), 5.63 (s, 1H), 3.79 (dt, *J* = 14.1, 6.6 Hz, 1H), 3.48 (brs, 1H), 3.34 (m, 1H), 3.02 (t, *J* = 6.6 Hz, 2H), 2.89 (s, 1H), 2.85 (d, *J* = 4.5 Hz, 1H), 2.23 (dd, *J* = 13.5, 7.2 Hz, 1H), 1.93 (m, 2H), 1.66 (m, 3H), 1.51–0.75 (m, 3H), 1.43 (s, 3H), 1.25 (d, *J* = 1.8 Hz, 3H), 1.09 (m, 3H), 0.78 (d, J = 6.9 Hz, 3H), 0.51 (s, 3H) ppm; ^13^C NMR (75 MHz, CDCl_3_) *δ* 176.2, 144.7, 136.3, 133.4, 132.2, 128.1, 127.5, 127.4, 127.2, 126.0, 125.4, 97.6, 73.8, 55.9, 55.7, 52.8, 45.3, 42.5, 40.0, 39.7, 39.5, 33.8, 32.9, 30.9, 27.8, 25.8, 22.4, 21.9, 18.4, 11.7, 1.0 ppm; HRMS: Calcd. for C_31_H_40_BrNNaO_2_ 560.2140, found 560.2143.

#### (3S,5S)-5-((R)-2-((1R,3aR,7aR,E)-4-(Bromomethylene)-7a-methyloctahydro-1H-inden-1-yl)propyl)-3-methyl-1-(2-(naphthalen-2-yl)ethyl)-3-((triethylsilyl)oxy)pyrrolidin-2-one (8)

To a solution of lactam **7** (42.3 mg, 0.0787 mmol) in CH_2_Cl_2_ (2.7 mL) was added 2,6-lutidine (0.11 mL, 0.944 mmol) and TESOTf (0.18 mL, 0.787 mmol) at 0°C under argon, and the mixture was stirred at the same temperature for 30 minutes. To the reaction mixture was added sat. NaHCO_3_ aq. (2.7 mL) and the organic layer was extracted with dichloromethane for three times. The combined organic layer was dried over MgSO_4_, filtered, and concentrated *in vacuo*. The residue was purified by column chromatography on silica gel (*n*-hexane/ethyl acetate = 30:1) to give **8** (29.7 mg, 58%).

Spectral data for **8**: [α]^25^_D_ = +115.0 (*c* 0.2, CHCl_3_); ^1^H NMR (300 MHz, CDCl_3_) *δ* 7.78 (m, 3H), 7.66 (brs, 1H), 7.44 (m, 2H), 7.35 (dd, *J* = 8.7, 1.5 Hz, 1H), 5.63 (s, 1H), 3.75 (dt, *J* = 13.8, 7.5 Hz, 1H), 3.56 (m, 1H), 3.34 (m, 1H), 3.00 (t, *J* = 7.4 Hz, 2H), 2.87 (m, 1H), 2.20 (dd, *J* = 13.2, 6.6 Hz, 1H), 1.94 (m, 2H), 1.66 (m, 4H), 1.51–0.75 (m, 10H), 1.43 (s, 3H), 0.94 (t, *J* = 7.8 Hz, 9H), 0.82 (d, *J* = 5.4 Hz, 3H), 0.62 (q, *J* = 7.8 Hz, 6H), 0.52 (s, 3H) ppm; ^13^C NMR (75 MHz, CDCl_3_) *δ* 174.9, 144.7, 136.6, 133.5, 132.2, 128.1, 127.6, 127.4, 127.3, 127.2, 126.0, 125.4, 97.6, 75.9, 56.1, 55.7, 52.4, 45.4, 43.6, 42.3, 39.7, 39.6, 34.1, 32.8, 30.9, 27.8, 25.6, 22.4, 21.9, 18.4, 11.8, 7.0, 6.0 ppm; HRMS: Calcd. for C_37_H_54_BrNaNO_2_Si 674.3005, found 674.2999.

#### 1α,25(OH)_2_ D_3_-26,23-Vitamin D lactam (DLAM)-2-2-Nap (2a)

To a solution of (23*S*,25*S*)-bromoolefin **8** (7.9 mg, 0.012 mmol), A-ring synthon of **9a** (7.9 mg, 0.015 mmol) and Et_3_N (0.20 mL) in toluene (0.20 mL) was added Pd(PPh_3_)_4_ (3.2 mg, 0.0028 mmol) at room temperature under argon, then the resulting mixture was heated at 90°C. After stirring for 1 h, the reaction mixture was concentrated *in vacuo*, and the residue was purified by column chromatography on silica gel (*n*-hexane/ethyl acetate = 60:1) to give coupling product (10.7 mg). To a solution of the coupling product (10.7 mg, 0.011 mmol) in MeOH (2.3 mL) was added MsOH (0.046 mL) at 0°C under argon, and the mixture was stirred at room temperature. After stirring for 1 h, the reaction was quenched with H_2_O, and the organic layer was extracted with chloroform for three times. The combined organic layer was dried over MgSO_4_, filtered, and concentrated *in vacuo*, and the residue was chromatographed on silica gel (chloroform/methanol = 90:1) to give **2a** (7.67 mg, >99%, 2 steps).

Spectral data for **2a**: [α]^25^_D_ = +6.7 (*c* 0.2, CHCl_3_); ^1^H NMR (300 MHz, CDCl_3_) *δ* 7.79 (m, 3H), 7.64 (brs, 1H), 7.45 (m, 2H), 7.36 (dd, *J* = 8.7, 1.8 Hz, 1H), 6.36 (d, *J* = 11.1 Hz, 1H), 6.00 (d, *J* = 11.1 Hz, 1H), 5.32 (s, 1H), 4.98 (s, 1H), 4.43 (q, *J* = 3.9 Hz, 1H), 4.23 (m, 1H), 3.80 (m, 7.0 Hz, 1H), 3.49 (s, 1H), 3.02 (m, 2H), 2.82 (dd, *J* = 13.8, 3.0 Hz, 1H), 2.60 (dd, *J* = 13.8, 3.0 Hz, 1H), 2.33 (m, 1H), 2.22 (m, 1H), 2.08–1.86 (m, 4H), 1.80–0.73 (m, 14H), 1.42 (s, 3H), 0.80 (d, *J* = 4.8 Hz, 3H), 0.50 (s, 3H) ppm; ^13^C NMR (125 MHz, CD_3_OD) *δ* 178.0, 149.8, 142.2, 138.1, 135.8, 135.1, 133.9, 129.2, 128.6, 128.6, 128.5, 127.1, 126.6, 124.8, 119.1, 112.0, 74.8, 71.4, 67.4, 58.1, 57.4, 54.6, 46.8, 46.1, 43.9, 43.7, 42.0, 41.8, 40.6, 34.9, 34.3, 30.8, 29.9, 28.8, 25.5, 24.5, 23.2, 18.8, 12.2 ppm; HRMS: Calcd. for C_39_H_51_NaNO_4_ 620.3716, found 620.3732.

#### 2α-Methyl-DLAM-2-2-Nap (2b)

To a solution of (23*S*,25*S*)-bromoolefin **8** (7.4 mg, 0.011 mmol), A-ring synthon of **9b** (5.2 mg, 0.014 mmol) and Et_3_N (0.19 mL) in toluene (0.19 mL) was added Pd(PPh_3_)_4_ (6.0 mg, 0.0052 mmol) at room temperature under argon, then the resulting mixture was heated at 90°C. After stirring for 1 h, the reaction mixture was concentrated *in vacuo*. The residue was chromatographed on silica gel (*n*-hexane/ethyl acetate = 80:1) to give coupling product (6.6 mg). To a solution of the coupling product (6.6 mg, 0.007 mmol) in MeOH (1.4 mL) was added MsOH (0.028 mL) at 0°C under argon, and the mixture was stirred at room temperature. After stirring for 1 h, the reaction was quenched with H_2_O, and the organic layer was extracted with chloroform for three times. The combined organic layer was dried over MgSO_4_, filtered, and concentrated *in vacuo*. The residue was chromatographed on silica gel (chloroform/methanol = 90:1) to give **2b** (3.7 mg, 55%, 2 steps).

Spectral data for **2b**: [α]^25^_D_ = +8.2 (*c* 0.3, CHCl_3_); ^1^H NMR (300 MHz, CDCl_3_) *δ* 7.80 (m, 3H), 7.65 (brs, 1H), 7.44 (m, 2H), 7.36 (dd, *J* = 8.4, 1.2 Hz, 1H), 6.38 (d, *J* = 11.4 Hz, 1H), 5.99 (d, *J* = 11.4 Hz, 1H), 5.28 (s, 1H), 5.00 (s, 1H), 4.31 (d, *J* = 3.9 Hz, 1H), 3.84 (m, 2H), 3.49 (s, 1H), 3.32 (m, 1H), 3.04 (brs, 2H), 2.83 (dd, *J* = 12.9, 3.0 Hz, 1H), 2.67 (dd, *J* = 12.9, 3.0 Hz, 1H), 2.26 (m, 3H), 1.94 (m, 5H), 1.74–0.85 (m, 10H), 1.42 (s, 3H), 1.07 (d, *J* = 6.9 Hz, 3H), 0.80 (d, *J* = 4.8 Hz, 3H), 0.50 (s, 3H) ppm; ^13^C NMR (125 MHz, CD_3_OD) *δ* 178.1, 149.0, 142.2, 138.1, 135.9, 135.1, 133.9, 129.3, 128.6, 127.1, 126.6, 124.7, 119.1, 113.2, 76.3, 74.8, 72.1, 71.5, 58.1, 57.4, 54.6, 46.8, 45.6, 44.7, 43.9, 42.0, 41.8, 40.6, 34.9, 34.3, 30.8, 29.9, 28.9, 25.5, 24.5, 23.2, 18.8, 13.3, 12.2, 9.2 ppm; HRMS: Calcd. for C_40_H_53_NaNO_4_ 684.3872, found 684.3852.

#### 2α-Hydroxypropyl-DLAM-2-2-Nap (2c)

To a solution of (23*S*,25*S*)-bromoolefin **8** (6.8 mg, 0.010 mmol), A-ring synthon of **9c** (6.8 mg, 0.013 mmol) and Et_3_N (0.17 mL) in toluene (0.17 mL) was added Pd (PPh_3_)_4_ (5.5 mg, 0.0048 mmol) at room temperature under argon, then the resulting mixture was heated at 90°C. After stirring for 1 h, the reaction mixture was concentrated *in vacuo*. The residue was chromatographed on silica gel (*n*-hexane/ethyl acetate = 25:1) to give coupling product (6.6 mg). To a solution of the coupling product (6.6 mg, 0.010 mmol) in MeOH (2 mL) was added MsOH (0.040 mL) at 0°C under argon, and the mixture was stirred at room temperature. After stirring for 1 h, the reaction was quenched with H_2_O, and the organic layer was extracted with chloroform for three times. The combined organic layer was dried over MgSO_4_, filtered, and concentrated *in vacuo*. The residue was chromatographed on silica gel (chloroform/methanol = 30:1) to give **2c** (3.8 mg, 58%, 2 steps).

Spectral data for **2c**: [α]^25^_D_ = +38.7 (*c* 0.1, CHCl_3_); ^1^H NMR (300 MHz, CDCl_3_) *δ* 7.79 (m, 3H), 7.65 (brs, 1H), 7.45 (m, 2H), 7.36 (dd, *J* = 8.4, 1.5 Hz, 1H), 6.39 (d, *J* = 11.1 Hz, 1H), 5.97 (d, *J* = 11.1 Hz, 1H), 5.27 (s, 1H), 4.99 (s, 1H), 4.39 (d, *J* = 2.7 Hz, 1H), 3.91 (m, 1H), 3.80 (q, *J* = 6.6 Hz, 1H), 3.70 (t, *J* = 5.7 Hz, 2H), 3.49 (s, 1H), 3.32 (m, 1H), 3.03 (m, 2H), 2.83 (dd, *J* = 13.5, 4.5 Hz, 1H), 2.66 (dd, *J* = 13.5, 4.5 Hz, 1H), 2.26 (m, 2H), 1.93 (t, *J* = 9.9 Hz, 2H), 1.74–0.85 (m, 18H), 1.42 (s, 3H), 0.80 (d, *J* = 4.8 Hz, 3H), 0.49 (s, 3H) ppm; ^13^C NMR (125 MHz, CD_3_OD) *δ* 178.1, 149.1, 142.3, 138.1, 135.7, 135.1, 133.9, 129.2, 128.6, 128.5, 127.1, 126.6, 124.7, 119.1, 113.4, 74.8, 74.0, 71.5, 70.7, 63.4, 58.1, 57.4, 54.6, 50.8, 46.8, 45.5, 43.9, 42.0, 41.8, 40.6, 34.8, 34.3, 31.3, 30.8, 29.9, 28.9, 25.5, 24.5, 23.9, 23.1, 18.8, 12.2 ppm; HRMS: Calcd. for C_42_H_57_NaNO_5_ 678.4134, found 678.4149.

#### 2α-Hydroxypropoxyl-DLAM-2-2-Nap (2d)

To a solution of (23*S*,25*S*)-bromoolefin **8** (6.1 mg, 0.0094 mmol), A-ring synthon of **9d** (6.3 mg, 0.011 mmol) and Et_3_N (0.15 mL) in toluene (0.15 mL) was added Pd (PPh_3_)_4_ (4.8 mg, 0.0041 mmol) at room temperature under argon, then the resulting mixture was heated at 90°C. After stirring for 1 h, the reaction mixture was concentrated *in vacuo*. The residue was chromatographed on silica gel (*n*-hexane/ethyl acetate = 20:1) to give coupling product (8.7 mg). To a solution of the coupling product (8.7 mg, 0.077 mmol) in MeOH (1.5 mL) was added MsOH (0.031 mL) at 0°C under argon, and the mixture was stirred at room temperature. After stirring for 1 h, the reaction mixture was quenched with H_2_O, and the organic layer was extracted with chloroform for three times. The combined organic layer was dried over MgSO_4_, filtered, and concentrated *in vacuo*. The residue was chromatographed on silica gel (chloroform/methanol = 30:1) to give **2d** (3.5 mg, 57%, 2 steps).

Spectral data for **2d**: [α]^25^_D_ = +37.8 (*c* 0.1, CHCl_3_); ^1^H NMR (300 MHz, CDCl_3_) *δ* 7.79 (m, 3H), 7.65 (brs, 1H), 7.46 (m, 2H), 7.36 (dd, *J* = 8.4, 1.5 Hz, 1H), 6.41 (d, *J* = 11.1 Hz, 1H), 5.99 (d, *J* = 11.1 Hz, 1H), 5.39 (s, 1H), 5.09 (s, 1H), 4.46 (brs, 1H), 4.07 (m, 1H), 3.84 (m, 4H), 3.51 (brs, 1H), 3.39 (m, 1H), 3.02 (brs, 2H), 2.82 (dd, *J* = 12.6, 5.1 Hz, 1H), 2.66 (dd, *J* = 13.5, 4.5 Hz, 1H), 2.23 (m, 2H), 2.00–0.82 (m, 20H), 1.42 (s, 3H), 0.79 (d, *J* = 4.8 Hz, 3H), 0.49 (s, 3H) ppm; ^13^C NMR (125 MHz, CD_3_OD) *δ* 178.1, 147.1, 142.7, 138.1, 135.1, 134.7, 133.9, 129.2, 128.6, 128.5, 127.1, 126.6, 125.1, 119.0, 114.7, 85.6, 74.8, 72.9, 69.3, 68.6, 60.4, 58.1, 57.4, 54.6, 46.9, 43.9, 42.3, 42.0, 41.8, 40.6, 34.8, 34.2, 33.6, 30.8, 30.5, 29.9, 28.8, 25.5, 24.5, 23.2, 18.8, 12.3 ppm; HRMS: Calcd. for C_42_H_57_NaNO_6_ 691.4084, found 691.4105.

## Supporting information

S1 Fig Spectra of synthesized compounds by NMR. (A-R) Each compound is depicted in the upper left in the panel.
(TIF)

S2 Fig Differential chromatin reorganization with different enrichment of transcription factor-binding sequences by DHT and Bicalutamide (Bic) in human prostate cancer cells (LNCaP cells). Assay for transposases-accessible chromatin (ATAC) analysis was performed in the LNCap cells, which were treated with DHT or Bic for 5 h prior to harvest. (A) Heatmap shows normalized ATAC signals around TSS and TES regions on the whole genome. Both ligands were potent to remodel chromatin accessibility. (B) The transcription factor-binding sequences in ATAC-seq peaks based on HOMER (http://homer.ucsd.edu/homer/) analysis in LNCaP cells with DHT or Bic for 4 h were searched and calculated. The top 10 motifs regulated by the lignads are shown. (C) Representative sequencing tracks for the gene KLK3 and RAD51AP1 loci show ATAC-Seq signals at the promoters and the known enhancers. The data were normalized and the scale on the y-axis was chosen for optimal visualization of peaks for each sample.
(TIF)

S3 Fig The gene ontology of the genes regulated by DHT and Bic. (A, B) List of the top genes for these AR ligands to make change of chromatin accessibility. (C) KEGG pathway analysis of the genes regulated by DHT or Bic.
(TIF)

S4 Fig Different profiles of chromatin accessibility regulated by DHT and Bic. (A) Venn diagram was used to assess chromatin accessibility profiles of AR ligand-regulated target regions measured by ATAC-Seq in LNCaP cells. Target regions regulated by DHT and Bic were selected with p-value<0.05 compared to MeOH which had more than 2-fold expression variations were extracted. (B) Venn diagram was used to detect up- or down-regulated effect of Bic for expression of the genes up-regulated by DHT.
(TIF)

S5 Fig Western blotting of VDR in the wild-type and VDR-KO HCT116 cells. Lack of VDR protein in the VDR KO HCT116 cells used in this study was confirmed by Western blotting. The left panel showed lack of VDR protein expression in the KO cells with the internal control of β-actin protein expression.
(TIF)

S6 Fig Representative sequencing track for the VDR target gene CYP24A1 by RNA-Seq signals in wild-type and VDR-KO HCT116 cells. The data that CYP24A1 induced by 1,25(OH)2D3 mediates VDR, confirmed CYP24A1 as the standard VDR target gene.
(TIF)

S7 Fig The original gel data and primer information. (A) Original gel data of Fig 3C. The area enclosed by the dotted line was used in the figure. (B) Primer information of qPCR and ChIP-qPCR data. (C) Original membrane data of S5 Fig. The area enclosed by the dotted line was used in the figure.
(PDF)

S1 Table List of the genes located on the opened chromatin regions by AR ligands. (XLSX)

S1 Data (ZIP)

S1 File (DOCX)
