# Supplementary figures and images for: Differential gene regulation by a synthetic vitamin D receptor ligand and active vitamin D in human cells
Source: PLoS One. 2023 Dec 13;18(12):e0295288. doi: 10.1371/journal.pone.0295288 (PMC10718451; doi:10.1371/journal.pone.0295288)

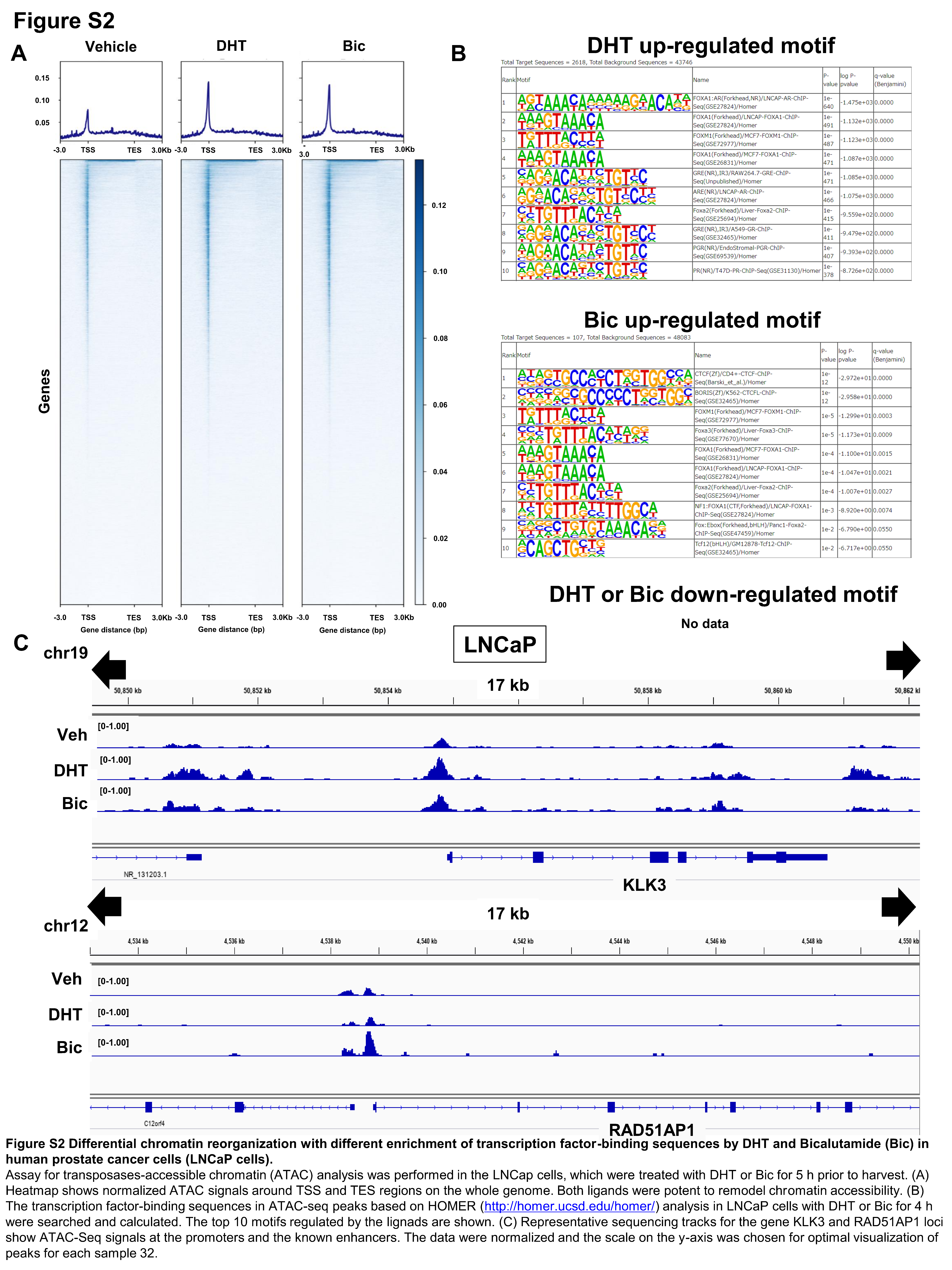

Supplement: S2 Fig — Assay for transposases-accessible chromatin (ATAC) analysis was performed in the LNCap cells, which were treated with DHT or Bic for 5 h prior to harvest. (A) Heatmap shows normalized ATAC signals around TSS and TES regions on the whole genome. Both ligands were potent to remodel chromatin accessibility. (B) The transcription factor-binding sequences in ATAC-seq peaks based on HOMER (http://homer.ucsd.edu/homer/) analysis in LNCaP cells with DHT or Bic for 4 h were searched and calculated. The top 10 motifs regulated by the lignads are shown. (C) Representative sequencing tracks for the gene KLK3 and RAD51AP1 loci show ATAC-Seq signals at the promoters and the known enhancers. The data were normalized and the scale on the y-axis was chosen for optimal visualization of peaks for each sample. (TIF) [file pone.0295288.s002.tif]

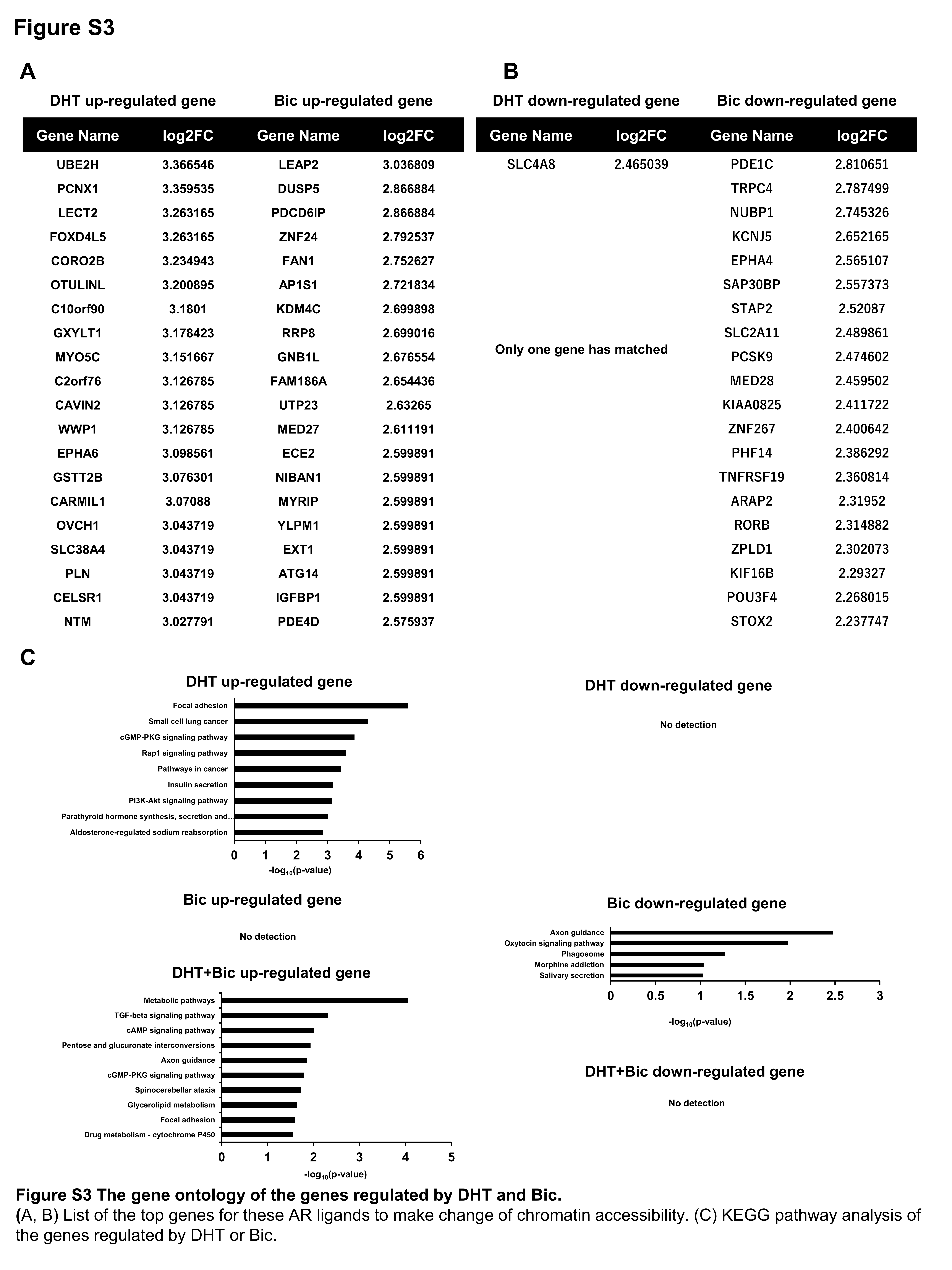

Supplement: S3 Fig — (A, B) List of the top genes for these AR ligands to make change of chromatin accessibility. (C) KEGG pathway analysis of the genes regulated by DHT or Bic. (TIF) [file pone.0295288.s003.tif]

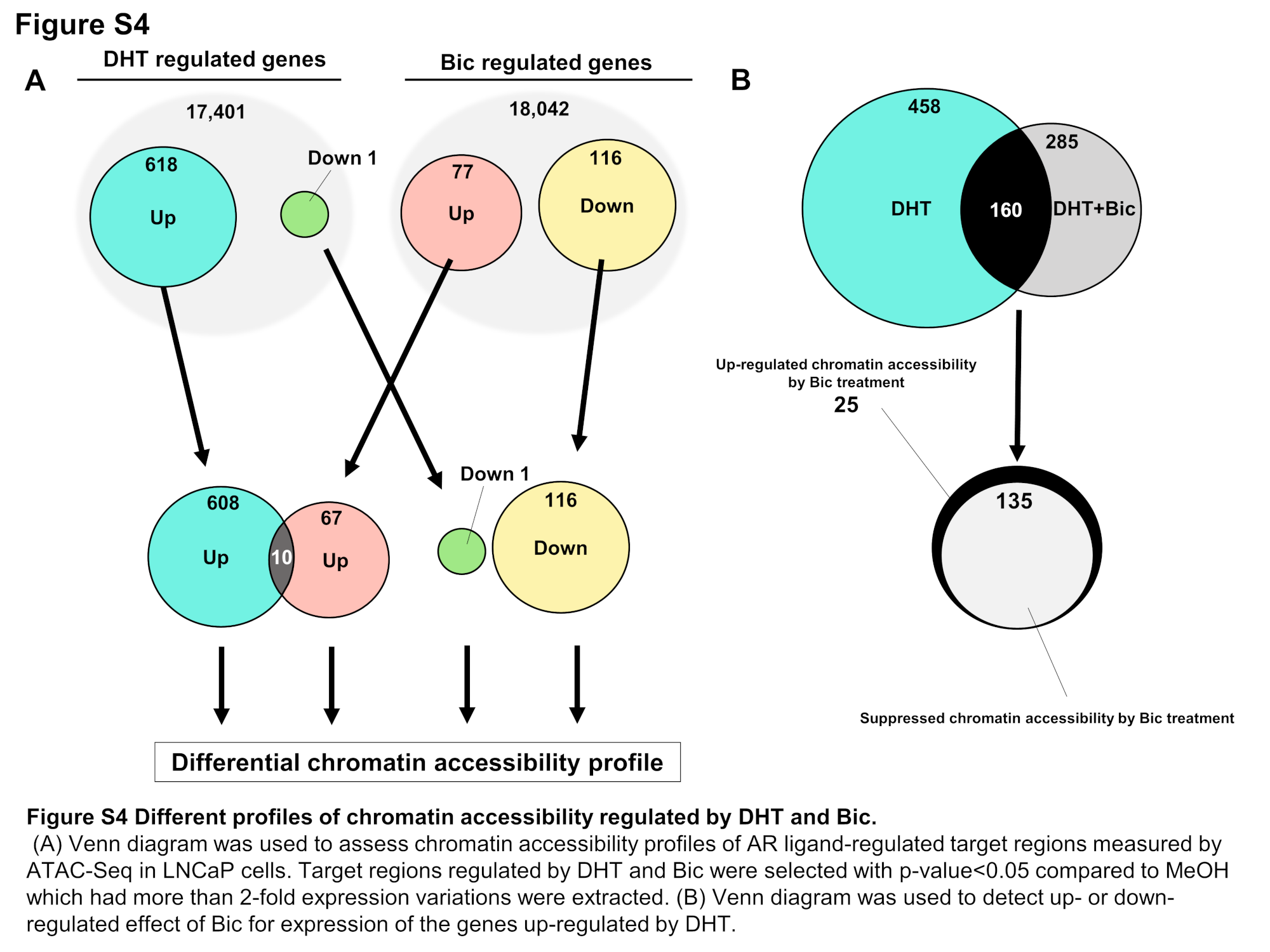

Supplement: S4 Fig — (A) Venn diagram was used to assess chromatin accessibility profiles of AR ligand-regulated target regions measured by ATAC-Seq in LNCaP cells. Target regions regulated by DHT and Bic were selected with p-value<0.05 compared to MeOH which had more than 2-fold expression variations were extracted. (B) Venn diagram was used to detect up- or down-regulated effect of Bic for expression of the genes up-regulated by DHT. (TIF) [file pone.0295288.s004.TIF]

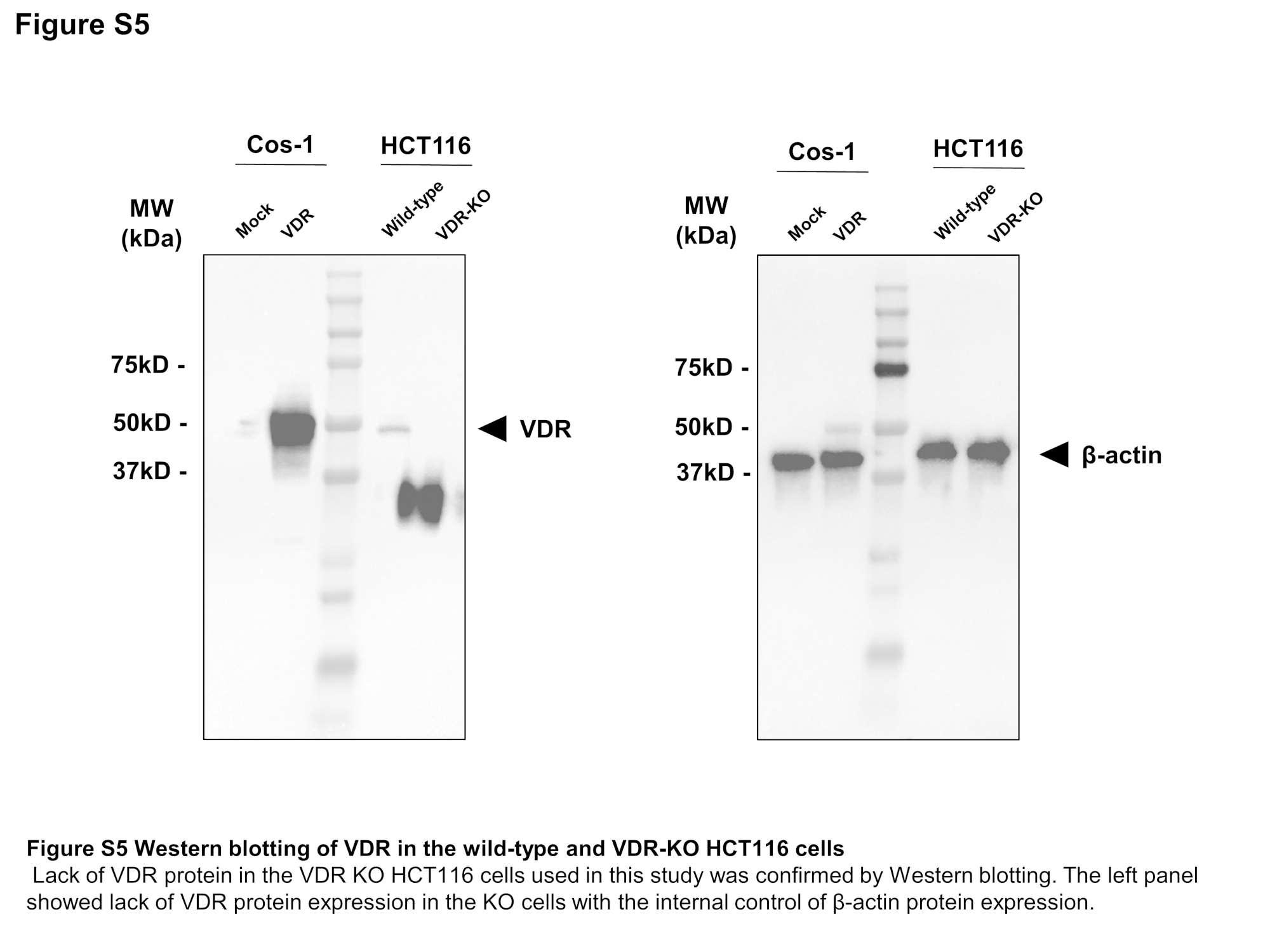

Supplement: S5 Fig — Lack of VDR protein in the VDR KO HCT116 cells used in this study was confirmed by Western blotting. The left panel showed lack of VDR protein expression in the KO cells with the internal control of β-actin protein expression. (TIF) [file pone.0295288.s005.TIF]

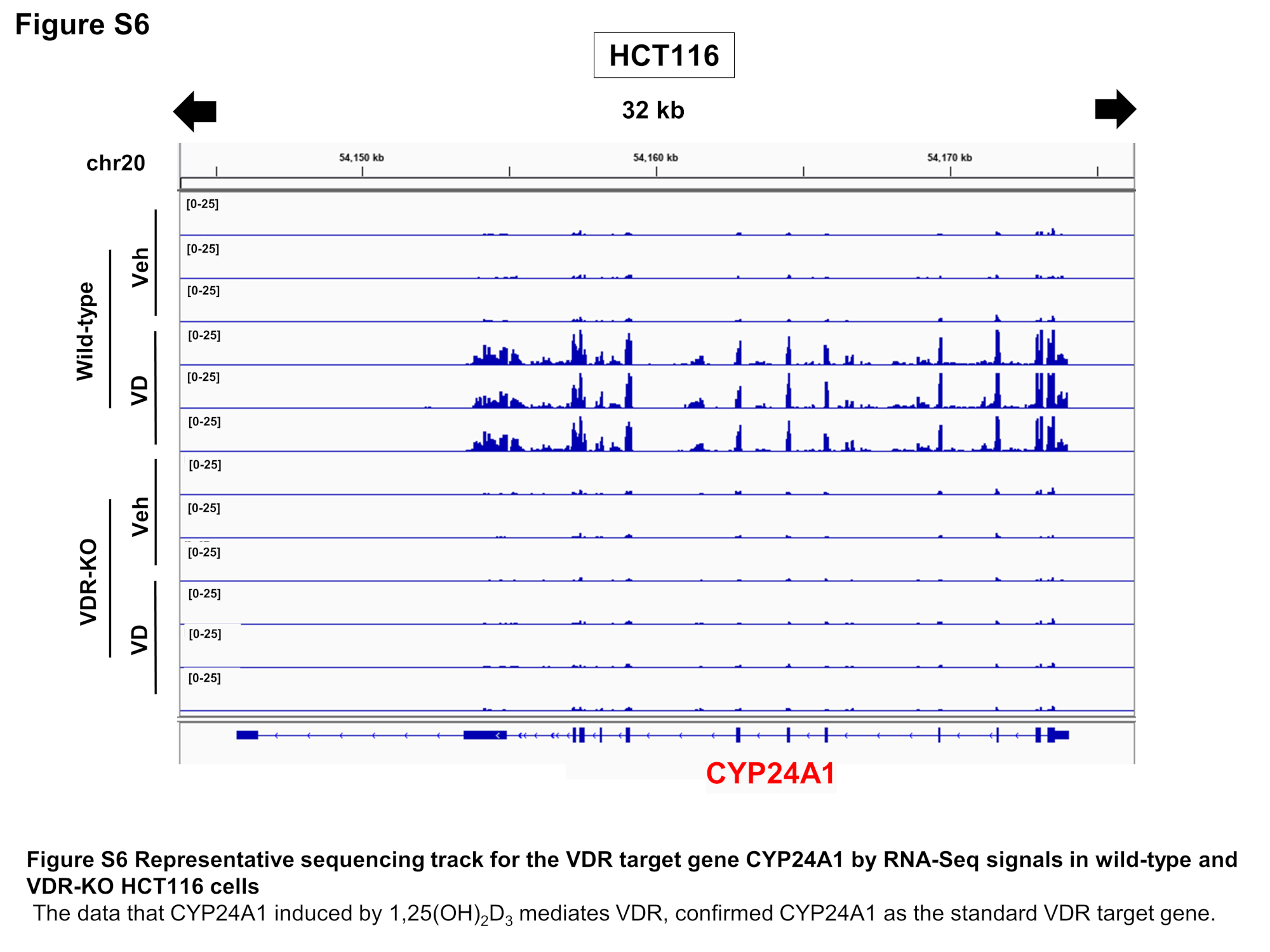

Supplement: S6 Fig — The data that CYP24A1 induced by 1,25(OH)2D3 mediates VDR, confirmed CYP24A1 as the standard VDR target gene. (TIF) [file pone.0295288.s006.TIF]
