# Supplementary material for: Differential gene regulation by a synthetic vitamin D receptor ligand and active vitamin D in human cells
Source: PLoS One. 2023 Dec 13;18(12):e0295288. doi: 10.1371/journal.pone.0295288 (PMC10718451; doi:10.1371/journal.pone.0295288)
Supplement: S7 Fig — (A) Original gel data of Fig 3C. The area enclosed by the dotted line was used in the figure. (B) Primer information of qPCR and ChIP-qPCR data. (C) Original membrane data of S5 Fig. The area enclosed by the dotted line was used in the figure. (PDF) [file pone.0295288.s007.pdf]

Figure S7

A

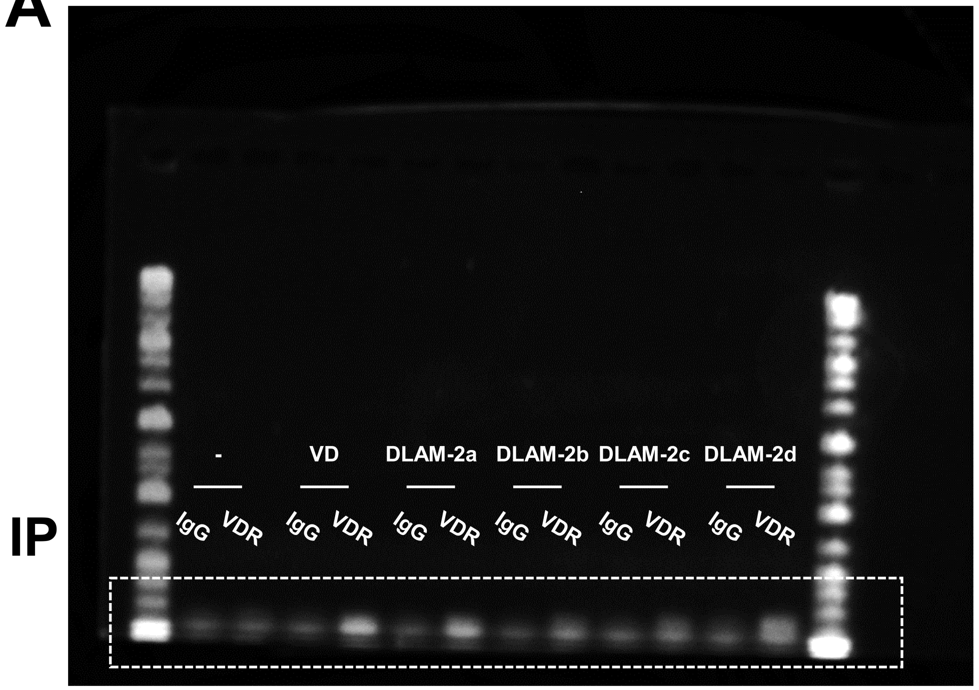

C

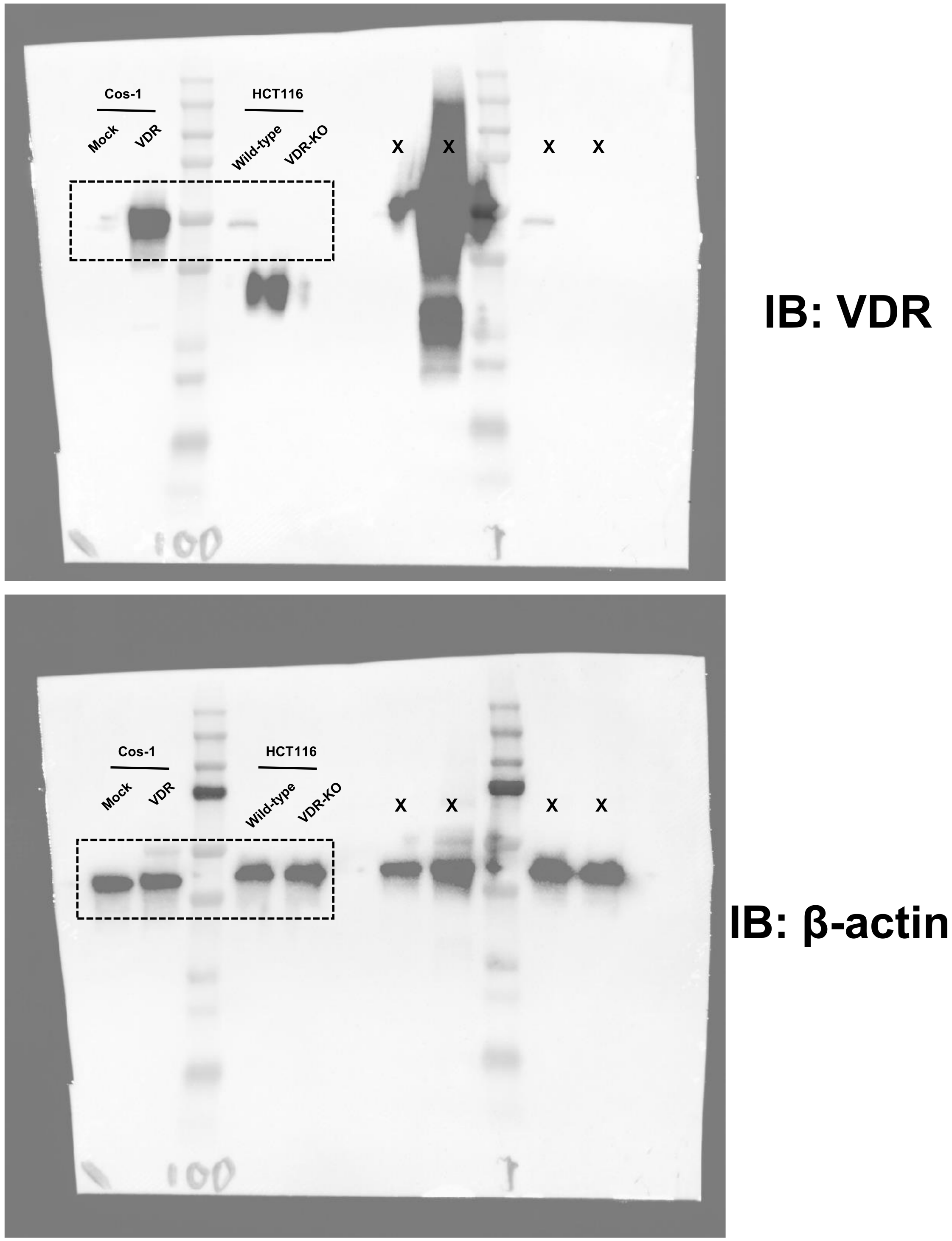

B

Primer	Sequence
β-actin-F	5'-ATTGGCAATGAGCGGTTC-3'
β-actin-R	5'-CGTGGATGCCACAGGACT-3'
CYP24A1-F	5'-CAGCGAACTGAACAAATGGTCG-3'
CYP24A1-R	5'-GCTCGACTGGAGTGACCATC-3'
VDRE-F	5'-CTTTGCTCAACAGGGACCTC-3'
VDRE-R	5'-AGACCTGCCACGTTAGTGCT-3'

Figure S7 The original gel data and primer information.
(A) Original gel data of Figure 3C. The area enclosed by the dotted line was used in the figure. (B) Primer information of qPCR and ChIP-qPCR data. (C) Original membrane data of Figure S5. The area enclosed by the dotted line was used in the figure.
