# Supplementary material for: Differential gene regulation by a synthetic vitamin D receptor ligand and active vitamin D in human cells
Source: PLoS One. 2023 Dec 13;18(12):e0295288. doi: 10.1371/journal.pone.0295288 (PMC10718451; doi:10.1371/journal.pone.0295288)
Supplement: S1 Data — (ZIP) [file pone.0295288.s009.zip › FID data for compounds 2a-d, 4-8/instruction.docx]

These FID files can be converted into NMR spectra by Delta 6.1 (JEOL, Tokyo, Japan).
