# Supplementary material for: Differential gene regulation by a synthetic vitamin D receptor ligand and active vitamin D in human cells
Source: PLoS One. 2023 Dec 13;18(12):e0295288. doi: 10.1371/journal.pone.0295288 (PMC10718451; doi:10.1371/journal.pone.0295288)
Supplement: S1 File — (DOCX) [file pone.0295288.s010.docx]

DOI List

Figure 1: 10.6084/m9.figshare.23806737

Figure 2: 10.6084/m9.figshare.23807478

Figure 3: 10.6084/m9.figshare.23807481

Figure 4: 10.6084/m9.figshare.23807487

Figure 5: 10.6084/m9.figshare.24716169

Figure 6: 10.6084/m9.figshare.23807712

Figure 7: 10.6084/m9.figshare.24716268

Figure 8: 10.6084/m9.figshare.24716277

S1 Figure: 10.6084/m9.figshare.24716280

S2 Figure: 10.6084/m9.figshare.24716286

S3 Figure: 10.6084/m9.figshare.24716289

S4 Figure: 10.6084/m9.figshare.23807763

S5 Figure: 10.6084/m9.figshare.23807766

S6 Figure: 10.6084/m9.figshare.23807787

S7 Figure: 10.6084/m9.figshare.24456142

S1 Table: 10.6084/m9.figshare.23807823
